# Structural insight into the inactivation of *Mycobacterium tuberculosis* non-classical transpeptidase Ldt_Mt2_ by biapenem and tebipenem

**DOI:** 10.1186/s12858-017-0082-4

**Published:** 2017-05-25

**Authors:** Mario A. Bianchet, Ying H. Pan, Leighanne A. Brammer Basta, Harry Saavedra, Evan P. Lloyd, Pankaj Kumar, Rohini Mattoo, Craig A. Townsend, Gyanu Lamichhane

**Affiliations:** 1 Department of Neurology, Johns Hopkins University School of Medicine, 725 N. Wolfe Street, Baltimore, MD 21205 USA; 2 Department of Biophysics and Biophysical Chemistry, Structural Enzymology and Thermodynamics Group, Johns Hopkins University School of Medicine, 725 N. Wolfe Street, Baltimore, MD 21205 USA; 3 Chemistry Department, United States Naval Academy, Annapolis, MD 21402 USA; 4 Department of Chemistry, Johns Hopkins University, Baltimore, MD 21218 USA; 5 Division of Infectious Diseases, Center for Tuberculosis Research, Taskforce to study Resistance Emergence & Antimicrobial development Technology (TREAT), Johns Hopkins University School of Medicine, Baltimore, MD 21231 USA; 6 Division of Infectious Diseases, Taskforce to study Resistance Emergence & Antimicrobial development Technology (TREAT), Johns Hopkins University School of Medicine, 1503 E. Jefferson Street, Baltimore, MD 21231 USA

**Keywords:** *Mycobacterium tuberculosis*, Carbapenem, L,D-transpeptidase, Enzyme inactivation, Peptidoglycan, Biapenem, Tebipenem

## Abstract

**Background:**

The carbapenem subclass of β-lactams is among the most potent antibiotics available today. Emerging evidence shows that, unlike other subclasses of β-lactams, carbapenems bind to and inhibit non-classical transpeptidases (L,D-transpeptidases) that generate 3 → 3 linkages in bacterial peptidoglycan. The carbapenems biapenem and tebipenem exhibit therapeutically valuable potencies against *Mycobacterium tuberculosis* (*Mtb*).

**Results:**

Here, we report the X-ray crystal structures of *Mtb* L,D-transpeptidase-2 (Ldt_Mt2_) complexed with biapenem or tebipenem. Despite significant variations in carbapenem sulfur side chains, biapenem and tebipenem ultimately form an identical adduct that docks to the outer cavity of Ldt_Mt2_. We propose that this common adduct is an enzyme catalyzed decomposition of the carbapenem adduct by a mechanism similar to S-conjugate elimination by β-lyases.

**Conclusion:**

The results presented here demonstrate biapenem and tebipenem bind to the outer cavity of Ldt_Mt2_, covalently inactivate the enzyme, and subsequently degrade via an S-conjugate elimination mechanism. We discuss structure based drug design based on the findings and propose that the S-conjugate elimination can be leveraged to design novel agents to deliver and locally release antimicrobial factors to act synergistically with the carbapenem carrier.

**Electronic supplementary material:**

The online version of this article (doi:10.1186/s12858-017-0082-4) contains supplementary material, which is available to authorized users.

## Background

Enzymes involved in the biosynthesis of peptidoglycan (PG) in bacteria have proven to be the Achille’s heel of bacteria; agents targeting this pathway (specifically the β-lactams and glycopeptides) represent some of most potent antibiotics in clinical medicine [1]. β-Lactam antibiotics inhibit these essential enzymes, causing pleiotropic toxicity of cellular physiology [2]. These antibiotics are the most extensively used class of antimicrobials in the world [1, 3]. However, this class of powerful drugs is seldom considered for treatment of tuberculosis, a major infectious disease with an annual human toll of ~9.6 million morbidity and ~1.5 million mortality globally [4]. The causative agent, *Mycobacterium tuberculosis* (*Mtb*), is historically considered to be naturally resistant to most β-lactams due to its chromosomally encoded β-lactamase that is capable of hydrolyzing penicillin and cephalosporin subclasses of β-lactams, and the potentially limited rate of diffusion of β-lactams across the lipid rich outer layer of the *Mtb* cell wall [5, 6].

The final step of PG biosynthesis in *Mtb* is catalyzed by two different classes of enzymes, namely the D,D-transpeptidases (also known as Penicillin Binding Proteins – PBPs) and L,D-transpeptidases (Ldts). D,D-Transpeptidases catalyze formation of the classical 4 → 3 linkages, where a transpeptide bond between the fourth amino acid (D-alanine) of one stem peptide and the third amino acid (*meso*-diaminopimelic acid, *m*DAP, in *Mtb*) of another is formed [1]. Conversely, Ldts catalyze the crosslinking of disaccharyl-tetrapeptide polymers by forming a transpeptide bond between the third amino acid in adjacent stem peptides thereby generating 3 → 3 crosslinkages between *m*DAP residues [7]. Although existence of these non-classical linkages in *Mtb* was first reported in 1974 [8], the enzymes responsible for generating them were only recently discovered [9, 10]. Approximately two-thirds of the linkages in *Mtb* PG are of 3 → 3 type and their synthesis is catalyzed by the Ldts, highlighting the importance of these non-classical transpeptidases to this pathogen [9, 11].

Emerging evidence suggests that carbapenems, a subclass of β-lactams, are not only poor substrates for β-lactamases, but are also uniquely able to inhibit Ldts and D,D-carboxypeptidases whose activities are vital for maintaining the physiology of *Mtb* PG [6, 9, 11–16]. In addition to the dominant Ldt, Ldt_Mt2_, *Mtb* possesses four additional sequence paralogs, which are aptly named Ldt_Mt1_, Ldt_Mt3_, Ldt_Mt4_, and Ldt_Mt5_ [10]. *Mtb* lacking Ldt_Mt2_ is severely attenuated for growth, virulence, has deformed cell walls and exhibits an increased susceptibility to β-lactams [10]. These phenotypes are further aggravated in *Mtb* lacking both Ldt_Mt2_ and Ldt_Mt1_ [17]. Loss of Ldt_Mt5_ compromises cell wall integrity leading to increased susceptibility to osmotic stress, crystal violet, and select carbapenems [15].

The structures of Ldt_Mt2_ bound to a PG stem fragment (PG-Ldt_Mt2_) [13] or meropenem [18–20], and very recently adducts with doripenem and a series of evolved carbapenems with new C2 side chains [21], and faropenem (a penem) [21, 22] have been reported. Structures of its paralogs, including *apo*-Ldt_Mt1_ and an imipenem-adduct structure of Ldt_Mt1_ [23], the *apo*- and meropenem-adduct structures of Ldt_Mt5_ [15] have also been reported. These data have provided structural and mechanistic insights about this family of enzymes, as well as details about the mechanisms of inactivation by carbapenem antibiotics.

The structure of the PG-Ldt_Mt2_ complex shows a dipeptide fragment of a PG stem peptide bound to the outer of two connecting cavities that flank the catalytic site coordinated by conserved residues of this Ldt family (aptly called Ldt motif: H*x*X_14-17_[S/T]HGC*h*N, where *x* represents a small residue (Gly, Ala, or Ser), X represents any residue, and *h* is any hydrophobic residue). The positioning of the bound PG fragment is consistent with a transpeptidation process in which donor and acceptor stem peptide substrates alternatively bind to the same outer cavity, and key equivalent D-alanyl groups are recognized by Ldt motif residues [13]. A transpeptidation process in which the donor and acceptor substrates enter the catalytic site from inner and outer cavities, respectively, has also been proposed [19].

Biapenem, an injectable, and tebipenem, an orally bioavailable carbapenem, are newer carbapenems with broad-spectrum activity and are often considered as the last resort to treat bacterial infections that are not amenable to other drug regimens. Both biapenem and tebipenem exhibit potent in vitro activity against *Mtb* [24, 25]. A recent report demonstrating efficacy of biapenem in a mouse model of tuberculosis [21] opens the possibility of repurposing carbapenems for the treatment of tuberculosis. In this study, we provide the molecular interactions of biapenem and tebipenem with Ldt_Mt2_ and propose a mechanism for the formation and decomposition of the adducts. We also discuss strategies for developing new antimicrobials by leveraging the carbapenem scaffold that inactivates Ldt_Mt2_.

## Methods

### General methods

Unless otherwise noted, all reagents were purchased from commercial sources. Primers were purchased from Integrated DNA Technologies. Biapenem and tebipenem (>98% purity) were purchased from Sigma-Aldrich. Molecular graphics and analyses were performed with the UCSF Chimera package [26] and the *Molecular Operating Environment (MOE) program* (v 2014.09; Chemical Computing Group Inc., 1010 Sherbooke St. West, Suite #910, Montreal, QC, Canada, H3A 2R7, 2014).

### Cloning, overexpression, and protein purification

Truncated versions of *ldt*_*Mt2*_ Δ(1–55) (encoding residues 56–408 of Ldt_Mt2_) were PCR amplified from *Mtb* H37Rv genomic DNA, digested with NdeI and XhoI and cloned into a modified pET28a vector as described [13]. The resulting vector was used to transform *E. coli* BL21 (DE3) cells (New England BioLabs). This strain was grown to A_600_ ~ 0.5 at 37 °C. The cultures were cooled to 16 °C, induced with 100 μM isopropyl 1-thio-β-d-galactopyranoside (IPTG), and growth was continued with shaking at 16 °C for 20 h. The cultures were then centrifuged at 3500 × g for 10 min at 4 °C and stored overnight at -20 °C. The pellets were thawed and resuspended in buffer containing 25 mM Tris, pH 8.0, 400 mM NaCl, 10% glycerol, 1 mM tris (2-carboxyethyl) phosphine (TCEP), and protease inhibitor cocktail (Roche). The cells were lysed by ultrasonication and centrifuged at 24,500 × g for 30 min at 4 °C. The supernatant was incubated with nickel-nitrilotriacetic acid (Ni-NTA) resin for 60 min at 4 °C. The resin was washed and His_6_-tagged Ldt_Mt2_ was eluted from the resin over a stepwise gradient of 20 mM to 500 mM imidazole. All the fractions containing His_6_-tagged Ldt_Mt2_ (as determined by SDS-PAGE) were combined and subjected to dialysis for 48 hours at 4 °C against 2 L of 25 mM Tris-HCl, pH 8.0, 100 mM NaCl, 10% glycerol, and 1 mM TCEP in the presence of TEV protease (1:100 TEV:Ldt_Mt2_). The dialysis buffer was replaced 3–4 times. The dialyzed protein was incubated with fresh Ni-NTA resin for 60 min at 4 °C and the flow-through containing Ldt_Mt2_ without the *N*-terminal His_6_ tag was collected. The presence and purity of the protein was confirmed by SDS-PAGE and its concentration was quantified using a Nano-drop™ spectrophotometer. Aliquots of Ldt_Mt2_ were flash frozen in liquid N_2_ and stored at -80 °C until further use.

### Calorimetry

The Δ(1–55) Ldt_Mt2_ protein and carbapenem stocks were thawed immediately before the experiment. The calorimetry buffer solution containing 25 mM Tris-HCl, 100 mM NaCl, 1 mM TCEP, pH 7.5 was prepared fresh prior to each experiment. Protein samples were prepared by buffer exchanging into the calorimetry buffer solution using a HiTrap (GE HealthCare) desalting column according to the manufacturer’s protocol. Carbapenem solutions were prepared in the calorimetry buffer as well. All solutions were filtered through a 0.2 μm filter and were degassed for 20 min with vigorous stirring. Enzyme concentration was determined by measuring the absorption at 280 nm and calculated using a calculated extinction coefficient [27] of 71,379 cm^-1^M^-1^. The heat exchange of the reaction between the carbapenem and the enzyme was measured following standard VP-ITC instrumental protocol. Equal amounts (10 μL) of either biapenem (620 μM) or tebipenem (1 mM) were injected with stirring into the calorimeter cell containing 1.4 mL of enzyme (46.4 μM for biapenem and 43 μM for tebipenem). Carbapenem injections into the cell containing Ldt_Mt2_ were performed with 2000 s or 1000 s equilibrations for biapenem and tebipenem, respectively, between injections until reaction completion. Experiments were carried out at 27 °C.

### Crystallization

Crystals of adducts of Ldt_Mt2_ with biapenem and tebipenem were obtained by the hanging-drop vapor diffusion method at 20 °C using Δ(1–55) Ltd_Mt2_ (12.8 mg/mL in 25 mM Tris-HCl pH 8.0, 100 mM NaCl, and 10% glycerol) incubated with 4 mM of the carbapenem. Drops (2 μL) of protein/carbapenem sample and 2 μL of reservoir solution were equilibrated against 500 μL of reservoir solution containing 18% v/v PEG mono-methyl ester 5000, 120 mM ammonium sulfate in 100 mM Tris-HCl buffer at pH 7.5. Two crystals from a previous crystallization were crushed in mother liquor and the resulting microseeds were used in a 1/100 dilution in a second optimization round under the same conditions. Crystals suitable for data collection grew within one week; 3-week old crystals were used in the data acquisition. Crystals of the *apo*-Ldt_Mt2_ were grown in the same condition and did not require seeding.

### Data collection, structure determination, and refinement

Diffraction data for the *apo* crystal were collected at the Berkeley National Laboratory Advanced Light Source (ALS) synchrotron beam-line B5.0.2. Diffraction data for the adduct-Ldt_Mt2_ crystals were collected in house at the Biophysical and Biophysical Chemistry Department X-ray Facility with a SATURN 944+ CCD detector using as source a Cu rotating anode X-ray generator FRE^+^ Superbright™ equipped with a Varimax™ optics (RIGAKU Inc, Texas). All the diffraction experiments were performed with crystals frozen in liquid nitrogen in their respective mother liquor with addition of 15% glycerol as cryoprotectant. The X-ray datasets were processed and scaled using the program HKL2000 [28]. The data collection statistics are summarized in Table 1. The crystal structure of Δ(1-55)Ltd_Mt2_ (*apo*-Ldt_Mt2_) was determined by molecular replacement with the program PHASER [29] using 3VYN [20] as initial model. After rigid body refinement of each separate domain (55–147, 148–251, and 252–408), the resulting model was subjected to cycles of coordinates, real space, isotropic B-factor, occupancy, and TLS refinement with the program PHENIX [30] followed by manual rebuild with the molecular modeling program Coot [31]. The adduct crystals were solved using the same protocol. RMSD calculations and alignments were performed using the MOE. Additional structural figures were drawn using MOE and Chimera, v.1.9 [26]. Coordinates and structure factors were deposited in the PDB under the codes 5D7H (*apo*-Ldt_Mt2_), 5DCC (Ldt_Mt2_-biapenem), and 5DC2 (Ldt_Mt2_-tebipenem).

**Table 1:** Data collection and refinement statistics

Crystals	Apo	Biapenem	Tebipenem
Source	ALS 5.0.2	Rotating anode	FRE^+^ Superbright™
Wavelength (Å)		1.5418	
Resolution range (Å)	48.62–2.49 (2.58–2.49)	37.02–2.18 (2.26–2.18)	43.16–2.45 (2.54–2.45)
Space group		P 1 2_1_1	
Unit cell	61.3 95.6 75.6 90 92.6 90	60.8 93.4 75.2 90 92.6 90	60.9 94.4 75.4 90 92.9 90
Total reflections	548790	1196329	570849
Unique reflections	29184 (2480)	42181 (3780)	31106 (2957)
Multiplicity	3.6 (2.8)	3.6 (2.9)	3.7 (2.9)
Completeness (%)	98.6 (85.2)	96.4 (87.2)	99.1 (94.4)
Mean I/sigma (I)	22.1 (2.1)	23.3 (4.25)	21.6 (3.45)
Wilson B-factor	47.76	28.13	26.49
R_merge_	0.085 (0.44)	0.06 (0.20)	0.07 (0.20)
R_work_	0.16 (0.24)	0.16 (0.18)	0.17 (0.21)
R_free_	0.22 (0.315)	0.21 (0.24)	0.21 (0.28)
N^o^ non-hydrogen atoms	5646	6214	6013
Protein	5358	5411	5402
Ligands	95	201	216
Water Molecules	193	602	395
Protein residues	702	705	707
RMS (bonds)	0.009	0.009	0.003
RMS (angles)	1.07	1.06	0.71
Ramachandran favored (%)	97	97.5	96.8
Ramachandran outliers (%)	0	0	0
Average B-factor (Å^2^)	45.5	32.7	30.6
Protein	44.8	30.7	26.4
MTOA (Occupancy)	—	42.5 (0.7)	56.2 (0.93)
Other Ligands (Occupancy)	88.4	69.75 (0.99)	62.5 (0.99)
Solvent	44.0	39.3	55.4

Diffraction and refinement statistics for *apo*-Ldt_Mt2_, Ldt_Mt2_-biapenem and Ldt_Mt2_-tebipenem complexes. Statistics for the highest-resolution shell are shown in parentheses

### ESI-TOF mass spectrometry analysis

Samples were analyzed by Ultra-performance liquid chromatography (UPLC)-high resolution MS with a Waters Acquity H-Class system utilizing a Waters Acquity BEH-300 UPLC column packed with a C_4_stationary phase (2.1 × 50 mm, 1.7 μm) in conjunction with HRMS analysis by a Waters Xero-G2 quadropole-TOF electrospray mass spectrometer. Proteins (2 μM) in 12.5 mM Tris-HCl buffer at pH 8 were incubated in the presence or absence of 50 μM carbapenem for 5 h at room temperature. Reactions were quenched by the addition of trifluoracetic acid (TFA, final concentration 0.1%). Samples were filtered through a 0.2 μm filter and analyzed by UPLC/MS at 60 °C. Mobile phase: 0–1 min 90% water + 10% acetonitrile (ACN) + 0.1% TFA, 1–7.5 min gradient up to 20% water + 80% ACN + 0.1% TFA, 7.5–8.4 min 20% water + 80% ACN + 0.1% TFA, 8.4–8.5 min gradient up to 90% water + 10% ACN + 0.1% TFA, 8.5–10 min 90% water + 10% ACN + 0.1% TFA. Flow rate = 0.3 mL min^-1^ T = 60 °C. The theoretical average mass including amino acids from the TEV cleavage site and linker of 38085 Da was calculated using the ExPASy mass calculator server (http://web.expasy.org/compute_pi/).

### MALDI-TOF mass spectrometry analysis

A 10 mg/ml sinapinic acid stock solution was prepared in 40% v/v ACN containing 0.1% TFA. The reaction sample was prepared using equimolar amounts of Ldt_Mt2_ and ligand (240 μM) in protein buffer (25 mM Tris, pH 8.0, 100 mM NaCl, 10% glycerol, and 1 mM TCEP). Aliquots of the reaction taken at 15, 40 and 24 h were diluted 5-fold in 40% ACN, then mixed with matrix stock solution and 0.5 μL was deposited onto the MALDI plate to dry. MALDI-TOF experiments were carried out on Voyager DE-STR (Applied Biosystems) at the JHUSOM mass spectrometry core facility and according to instrumental protocols. Data were processed using the Data Explore program. The mass gains as difference between the principal peak and that from the *apo*-Ldt_Mt2_ sample were calculated from the spectra.

### Carbapenem docking studies

The docking of biapenem and tebipenem molecules to the Ldt_Mt2_ outer cavity was simulated using the program MOE. Models of the carbapenems were built with the molecular builder feature of MOE using a small-molecule (MMX4) force field as is implemented in the program. These initial models were used as starting point of a quantum mechanical optimization using the program GAMESS [32] with a 6311+ Gaussian basis and a B3LYP density functional. Flexible docking of the carbapenems were performed utilizing the rigid body docking routine of MOE. An induced fit option using a weak harmonic tether (target distance 3.6 Å and harmonic constant of 0.5 kcal/mol) between the sulfur atom of Cys^354^ and C7 of the carbapenem as restrain was used to steer the docking. An AMBER12HT force field included in the software was used for the binding pocket minimization.

## Results

### Biapenem- and tebipenem-Ldt_Mt2_ adducts

**Fig. 1:**
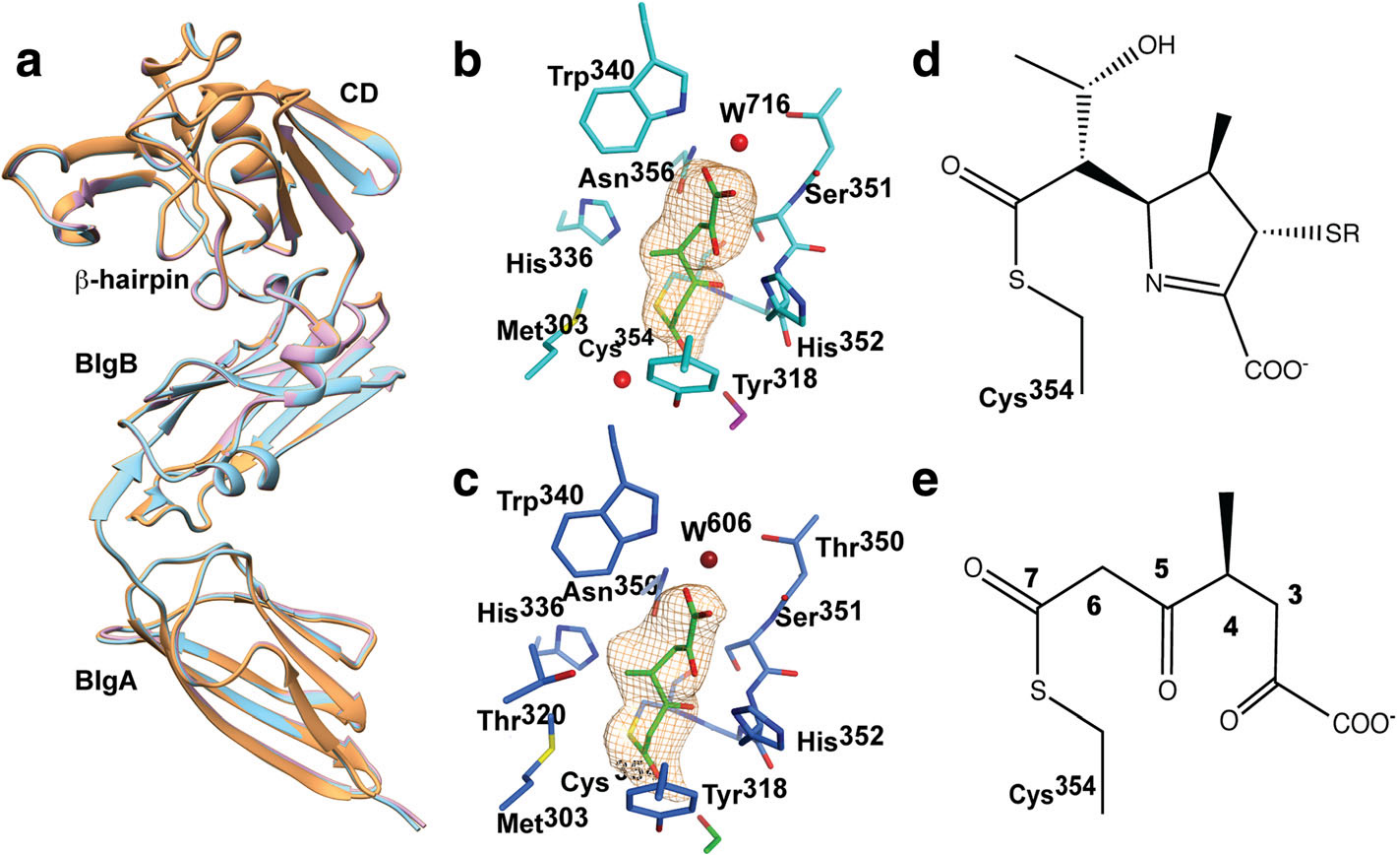
A*po*-Ldt_Mt2_ and adduct structures. **a** Overlap of the *apo-* (*brown*), biapenem- (*pink*), and tebipenem-complexes (*cyan*) of Ldt_Mt2_. BlgA (aa 56–150) and BlgB (aa 150–250) are Bacterial Immunoglobulin-like (BIg) domains and CD (aa 251–408) is the catalytic domain. **b** & **c** Simulating annealing omit map at the active site of Ldt_Mt2_-biapenem (**b**) and Ldt_Mt2_-tebipenem (**c**). The refined structure of the respective catalytic site and adduct are shown. The adduct atoms were omitted during a mock refinement cycle including a torsional simulated annealing step using the refinement program PHENIX. The omit maps were contoured at 3.5 σ level. In both panels the MTOA-adducts are colored green (stereo views in Additional file 1). **d** Chemical structure of the common β-lactam core of the anticipated non-degraded adducts. **e** Chemical structure of the observed adduct in both complexes

In this study, we produced crystals of Ldt_Mt2_ *apo* and enzyme reacted with biapenem or tebipenem, and collected X-ray diffraction data at 2.49, 2.18 and 2.45 Å resolutions, respectively. All crystal forms belong to the monoclinic P2_1_ space group with similar cell dimensions and contain two molecules per asymmetric unit (Table 1). Electronic density for the amino acids 56 to 407 of chain A and to the *C*-terminus (amino acid 408) of chain B was observed in all three structures. The structures were refined to a better than 0.17 R_work_ and 0.22 R_free_ with similar refinement protocols. All the structures have more than 96% of the residues in favored regions of their respective Ramachandran plots (Table 1). Our *apo*-Ldt_Mt2_ structure and the previously solved *apo*-Ldt_Mt2_ structure (3VYN [20]) have 0.7 Å of RMSD among 347 Cα atoms aligned. The *apo* and *holo* structures show negligible differences among them (Fig. 1a). The RMSDs are 0.4 and 0.3 Å between *apo*-Ldt_Mt2_ and Ldt_Mt2_-biapenem or Ldt_Mt2_-tebipenem, respectively, and 0.17 Å between the *holo* structures for the same 702 aligned Cα atoms aligned.

### Acyl adduct in Ldt_Mt2_-biapenem and Ldt_Mt2_-tebipenem complexes are identical

**Fig. 2:**
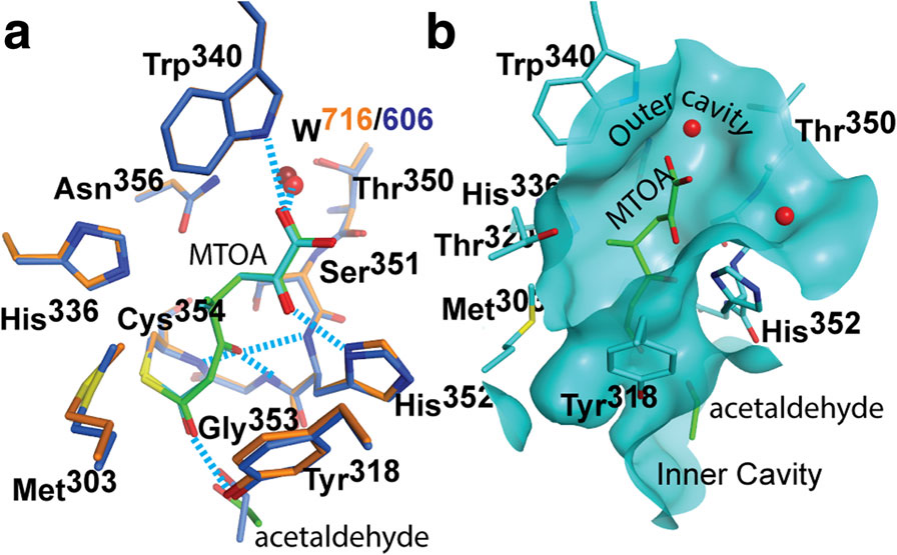
Adducts bound to the outer cavity of the Ldt_Mt2_ catalytic site. **a** Overlay of the Ldt_Mt2_-biapenem (enzyme carbon atoms colored in *blue*) and Ldt_Mt2_-tebipenem (in *orange*) catalytic sites as viewed from the outer cavity. The protein residues that interact with the MTOA adducts are shown and common hydrogen bond interactions indicated as cyan dashed lines. **b** Similar view of the catalytic site of the tebipenem complex crystal structure showing a portion of the solvent accessible surface of the outer cavity and tunnel connecting to the inner cavity, displaying the adduct and residues of the Ldt motif participating in the interaction with the it. The MTOA adduct is colored green

**Fig. 3:**
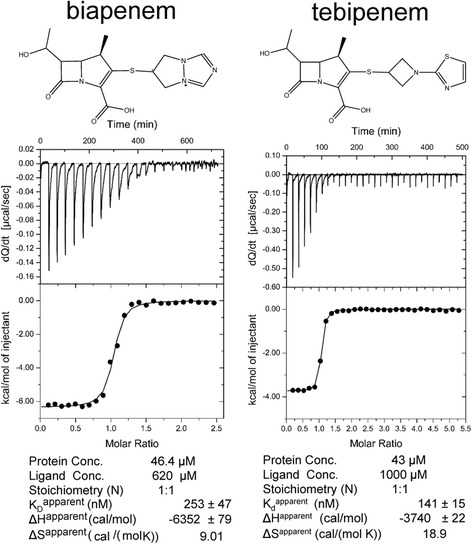
Heat exchange during biapenem and tebipenem adduct formation with Ldt_Mt2_. Both carbapenem show exothermic heat exchanges after the addition of the carbapenem to the reaction cell containing enzyme

**Fig. 4:**
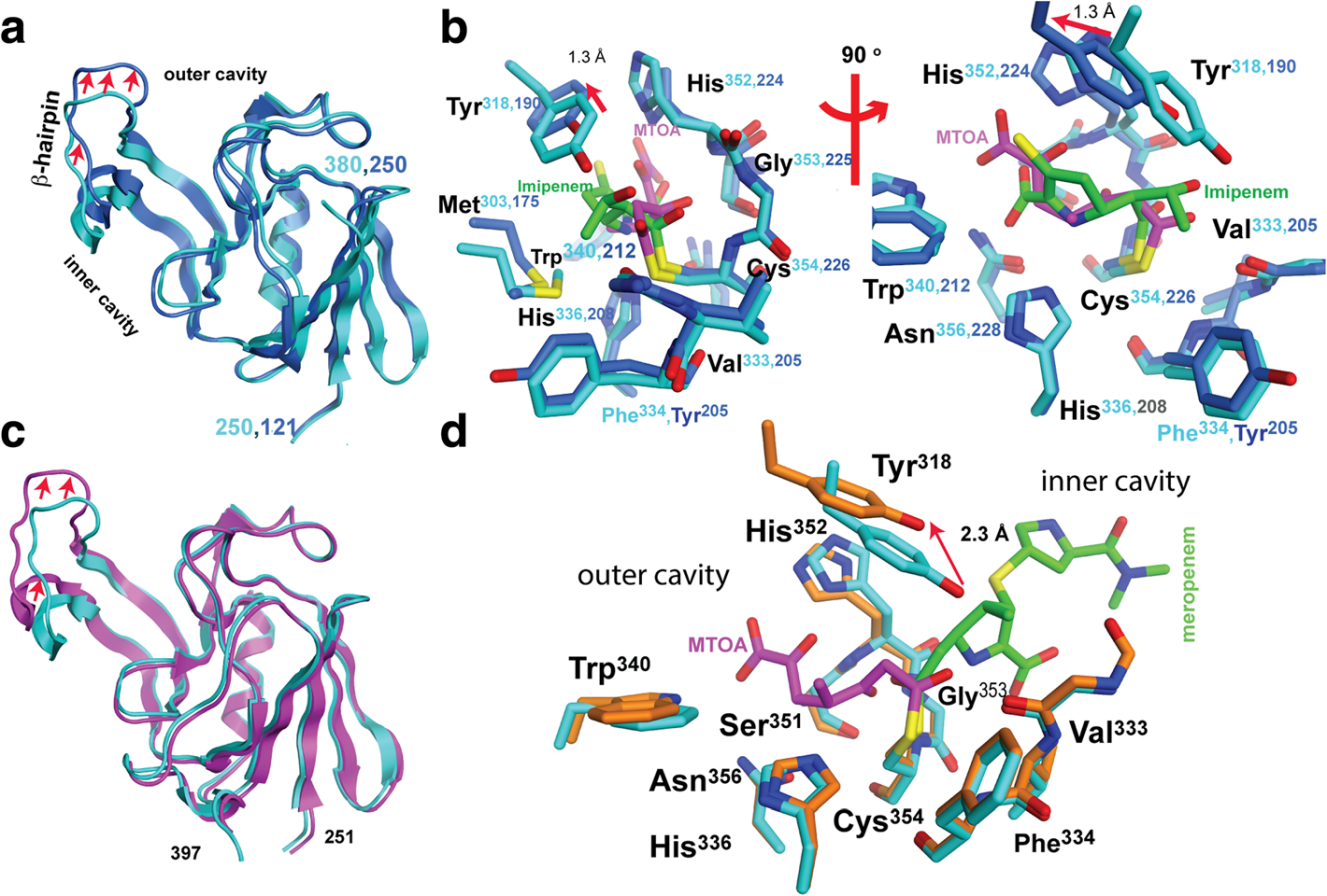
Structural comparison of *Mtb* Ldts with different carbapenem adducts. **a** Overlay of catalytic domains of *apo*-Ldt_Mt1_ (*colored blue*) and *apo*-Ldt_Mt2_ (*colored cyan*). *Red arrows* show the displacement of the β-hairpin flap between paralogs. **b** Two views related by a counterclockwise 90° rotation of the overlay of outer cavity-bound adducts of Ldt_Mt1_-imipenem (4VYM; carbon atoms of enzyme and adduct are colored *blue* and *green*, respectively) and Ldt_Mt2_-MTOA corresponding to the biapenem/Ldt_Mt2_ complex structure (colored in *cyan* and *magenta*). **c** Overlay of the Ldt_Mt2_ catalytic domains of Ldt_Mt2_-meropenem (3VYO; *magenta*) and Ldt_Mt2_-MTOA adduct (*cyan*). *Red arrows* show the displacement of the β-hairpin flap between these complexes forming inner- and outer-cavity adducts, respectively. **d** Overlay of the catalytic site of Ldt_Mt2_-meropenem (carbon atoms of the enzyme and the adduct are colored *orange* and *green*, respectively) and Ldt_Mt2_-MTOA (*cyan* and *magenta*). *Red arrows* in the panels (**b**) and (**d**) show the displacement of the conserved Tyr^318^ on the β-hairpin flap that in Ldt_Mt1_-imipenem and Ldt_Mt2_-meropenem interacts with the carbapenems hydroxyacetyl substituent, such interaction is loss in MTOA by the elimination of this group after adduct formation

Additional electron density connected to the catalytic Cys^354^ thiol group and extending to the outer cavity was observed in Ldt_Mt2_–biapenem and Ldt_Mt2_–tebipenem complexes. Interestingly, the electron densities in these complexes are similar in size and shape (Fig. 1b-c and Additional file 1: Figure S1a-b). The observed densities are larger than the glycerol molecule observed in the *apo*-Ldt_Mt2_ crystal form (Additional file 1: Figure S1c) but smaller than expected for an intact biapenem or tebipenem adduct as illustrated in Fig. 1d. In both adduct crystals (but not in the *apo*-crystal), a small-disconnected electron density was observed at the inner cavity near the catalytic cysteine. This density is inconsistent with any of the mother-liquor components and was therefore interpreted as a remnant of the hydroxyethyl substituent at C6 that is dissociated from the carbapenems (Additional file 1: Figure S1d). Based on the shape and coordination of the substituent groups and considering feasible chemical reactions (see below) at the carbapenem core (Fig. 1d), a (*S*)-4-methyl-2,5,7-trioxoheptanoic acid (MTOA) acyl adduct (Fig. 1e) was modeled bound to thiol group of the catalytic cysteine in both crystal forms. The glycerol molecule found in the *apo*-Ldt_Mt2_ crystal form is disconnected at 2.7 Å from the catalytic cysteine and poorly overlapping with the larger and connected MTOA adduct (Additional file 1: Figure S1e). The possibility of the enzyme reacting with a common contaminant product of the carbapenems degradation was also disregarded following AP-MALDI analysis of the reagents used (Additional file 1: Figure S2).

Both adducts are bound at the outer cavity in a hydrophilic cleft lined by the “oxyanion hole” (residues, His^336^, Asn^356^, His^352^ and Trp^340^) forming numerous hydrogen bonds with groups of the enzyme (Fig. 2). The carbonyl substituent at C7 forms a hydrogen bond with Tyr^318^ in the β-hairpin loop (aa 300–323). The carboxyl substituent at C5 docks in a pocket and accepts hydrogen bonds from the NH groups of His^352^, Gly^353^, and Cys^354^ (Fig. 2a). A structural water molecule (W716 in Ldt_Mt2_-biapenem and W606 in Ldt_Mt2_-tebipenem) coordinated by Trp^340^, Thr^350^, and Asn^356^ hydrogen bonds to the adduct carboxylate. The outer cavity, where the carbapenem binds, is accessible to solvent (Fig. 2b).

### Biapenem and tebipenem react exothermically with Ldt_Mt2_

The heat of reaction associated with acylation of Ldt_Mt2_ by biapenem and tebipenem was measured using a VP-ITC calorimeter (Fig. 3). Following injection, a fast exothermic reaction peak is observed when Ldt_Mt2_ is titrated with biapenem or tebipenem. In the case of biapenem, the sharp initial heat exchange peak was followed by a very slow return to the baseline indicating that a secondary reaction (or reactions) follows the initial rapid binding/acylation (Fig. 3). Although the adduct formation does not involves a binding equilibrium, the apparent thermodynamic parameters of binding are shown in Fig. 3.

### Mass spectrometry confirms the acylation of Ldt_Mt2_ by the carbapenems

Following a five hour incubation of Ldt_Mt2_ with molar excess of the carbapenems (1:25, enzyme:carbapenem ratio) the mass spectra of the reacted species were analyzed (Table 2; Additional file 1: Figure S3). Despite only the MTOA adduct (Δm/z of 184 Da) being observed in the crystal structure, the tebipenem-Ldt_Mt2_ sample shows peaks with a Δm/z of 384.5 Da, corresponding to the expected mass of an intact tebipenem adduct to Ldt_Mt2_ (Δm/z of 384 Da), with a Δm/z of 339.5 Da, and a minor peak with a Δm/z of 32 Da (Table 2). This discrepancy is likely attributable to the long incubation times required during co-crystallization. The second peak has a 45 Da decrease with respect to the largest one. The smallest peak of Δm/z = 32 Da may be the result of the irreversible oxidation of the thiol to sulphinic acid, resulting in a likely inactive form of the enzyme. Surprisingly, when biapenem was incubated with Ldt_Mt2_, a unique peak of Δm/z of 139.5 Da was observed (Additional file 1: Figure S3a).

**Table 2:** UPLC-HRMS of Inhibited Ld_Mt2_ species

m/z (Da)	Species	Intensity from the maximum %
(56-408)Ldt_Mt2_ (exact mass Mn = 38085.5 Da)		
38085.5	[Mn + H]^+^	100%
biapenem (exact mass = 352.1 Da)		
38225.0	[Mn + 139.5 + H]^+^	100%
tebipenem (exact mass = 384.1 Da)		
38469.6	[Mn + 384 + H]^+^	44%
38425.0	[Mn + 339 + H]^+^	39%
38117.0	[Mn + 32 + H]^+^	13%

M/z for native Ldt_Mt2_ and its species inactivated by biapenem and tebipenem. The exact mass for *apo*-Ldt_Mt2_ was calculated using ExPASy MW server. See also in Additional file 1: Figure S2 for the mass spectra

### The initial adducts degrade after acylation

We used MALDI-TOF mass spectrometry (Table 3, Additional file 1: Figure S4) to qualitatively investigate temporal changes in the mass of the adduct species in the secondary reaction observed by calorimetry toward identifying the degradation intermediates. After a short incubation, the difference between primary peaks of the mass spectra of unreacted Ldt_Mt2_ and the biapenem-Ldt_Mt2_ complex was Δm/z = 225 Da, which is less than the expected increase for an intact biapenem adduct (Δm/z = 352 Da). After 40 min and up to 24 h, the primary signal differences stabilized at 180 Da (45 Da less than the difference observed after a short incubation). In the tebipenem case, the primary peak differences after 40 min and 24 h of incubation showed Δm/z of 345 Da.

**Table 3:** Difference between primary peaks of the MALDI/TOF spectra of *apo*-Ldt_Mt2_ and Ldt_Mt2_ inactivated by biapenem and tebipenem at different reaction times

Δm/z (Da)	Incubation time
Ldt_Mt2_-biapenem	
225	15 min
180	40 min
Ldt_Mt2_-tebipenem	
345	40 min
345	24 h

See also in Additional file 1: Figure S4 for the experimental spectra

### Outer-cavity carbapenem adducts of Ldt_Mt1_ and Ldt_Mt2_ engage the Ldt motif

The catalytic domains (CDs) of *apo*-Ldt_Mt1_ (4JMN; aa 122–250)) and *apo*-Ldt_Mt2_ (aa 251–408) overlap with an RMSD of 1.1 Å for 129 Cα-atoms aligned; only the β-hairpin flap (Ldt_Mt1_, aa 172–195; Ldt_Mt2_, aa 300–323) is displaced in Ldt_Mt1_, with displacements ranging from 1.3 to 3.0 Å toward the outer cavity (Fig. 4a) that results in a 1.3 Å shift of Tyr^190^ from Tyr^318^ of Ldt_Mt2_ (Fig. 4b). Most of the residues lining the outer cavity are conserved between Ldt_Mt1_ and Ldt_Mt2_, however adduct-enzyme interactions of Ldt_Mt2_-biapenem and Ldt_Mt2_-tebipenem do not parallel those in Ldt_Mt1_-imipenem (Fig. 4b). The Ldt_Mt1_-imipenem (PDB ID = 4JMX) structure shows a partial imipenem molecule (the 2-thiol substituent is not observed) covalently bound to the catalytic cysteine (Cys^226^) and extending to the outer cavity of Ldt_Mt1_ [23]. Structures of *apo*- and *holo-*Ldt_Mt1_ are not significantly different following adduct formation (RMSD of 0.2 Å for 216 Cα-atoms aligned). The shift of Tyr^190^ in Ldt_Mt1_ increases the clearance for the hydroxyethyl side chain of imipenem (Fig. 4b). Conversely, in the Ldt_Mt2_-meropenem (PDB ID 3VYP [20]) and Ldt_Mt5_-meropenem structures (PDB ID 4ZFQ [15]) the meropenem adduct is bound at the inner cavity. In the Ldt_Mt2_-meropenem structure, meropenem binding shifts the β-hairpin flap between 2.0 and 4.5 Å away from the core of the CD (Fig. 4c). The meropenem adduct interacts minimally with motif residues and residues in the inner cavity pocket (Fig. 4d).

### The intact carbapenems dock at the outer cavity engaging Ldt motif residues

**Fig. 5:**
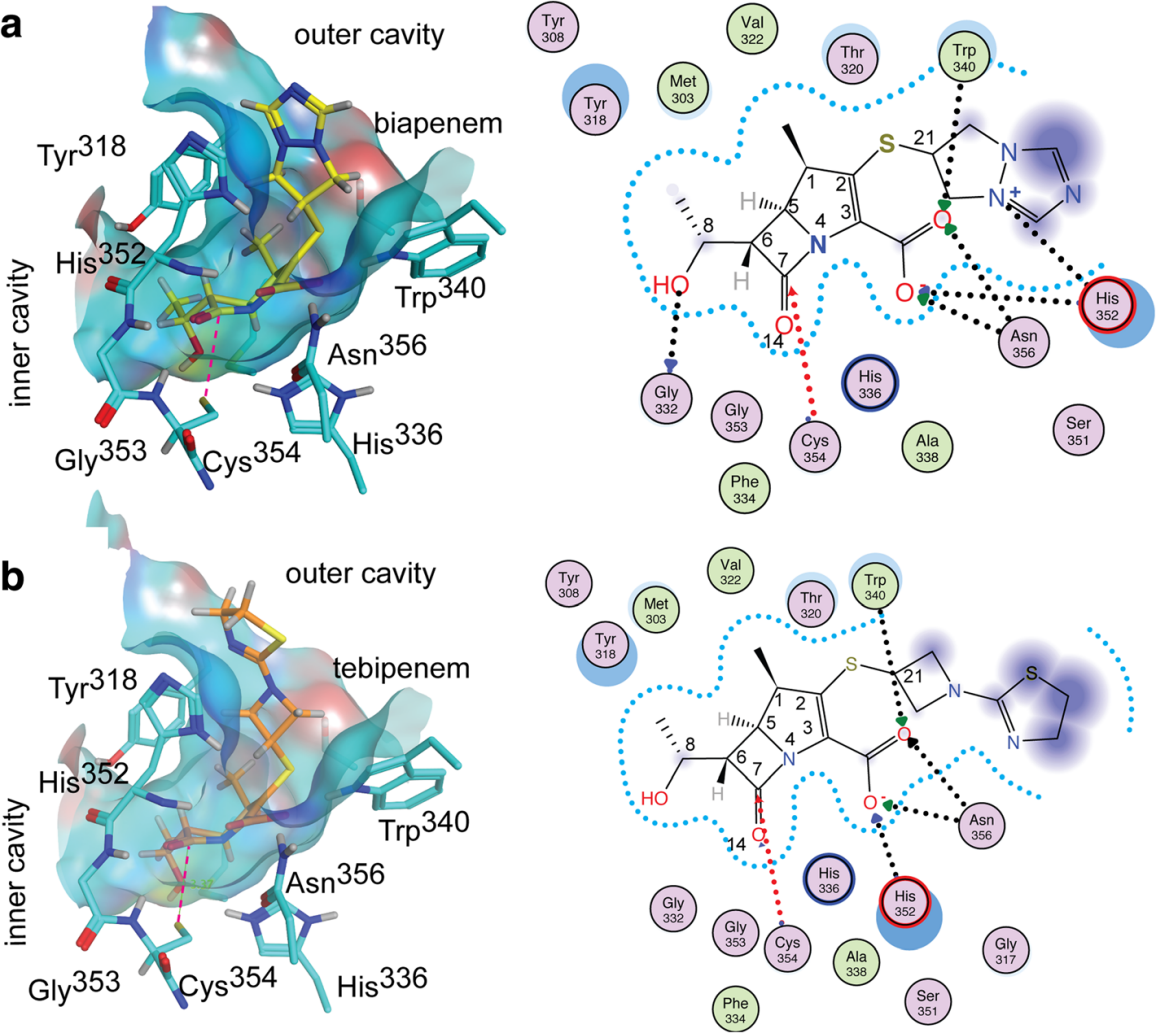
Predicted binding models of the biapenem and tebipenem interactions with Ldt_Mt2_. Docking results of the binding of intact (**a**) biapenem and (**b**) tebipenem to the outer cavity of Ldt_Mt2_. Left panels of (**a**) and (**b**) show the solvent accessible surface corresponding to the outer cavity and tunnel connecting to the inner cavity. The carbapenems (biapenem carbon atoms are *yellow*; tebipenem carbon atoms are *orange*) and Ldt_Mt2_ residues that participate in binding (*cyan*). The protein-ligand interactions are shown in the right panels. Residues circled are in Van der Waals contact with the ligand; those colored *green* and *pink* are hydrophobic and hydrophilic residues, respectively. Hydrogen bonds are marked as *black dashed arrows* starting in the proton donor. The *red dashed arrow* highlights the Cys^354^-C7 tether used for the steered docking simulation. Purple clouds around carbapenem atoms indicate solvent exposure, and the size of the clouds indicates the degree of exposure. Offset blue circles indicate partial exposure of the protein residue. The drawing and analysis were performed using MOE

Docking simulations of biapenem and tebipenem at the catalytic site of Ldt_Mt2_ favors extensive interactions of cyclic portions of the carbapenems with residues of the outer cavity (Fig. 5). When docked to the outer cavity, the portions of intact biapenem and tebipenem that mimic the D-Ala^4^ moiety of the acyl-donor/acceptor are strongly recognized by conserved outer cavity residues. The best scored docking positions of these carbapenems place the protonated Nε of the catalytic His^336^ (which deprotonates the catalytic Cys^354^ thiol thereby activating it) buried and in close contact (4.0 Å) with the C5 and N4 groups of these carbapenems. The carbapenem carboxylate accepts hydrogen bonds from His^352^, Asn^356^, and Trp^340^. The main-chain NH groups of His^352^, Gly^353^ and Cys^354^ (oxyanion hole) surround the carbonyl oxygen (O14) of the β-lactam ring. The hydrophobic side of the carbapenem core contacts aromatic ring of Tyr^318^ and Thr^320^. The hydroxyethyl substituent at C6 contacts Met^303^, Thr^320^, and His^352^, and hydrogen-bond to Tyr^318^. The hydrophilic residues Thr^320^, His^352^, and Tyr^318^ remain connected to bulk solvent and may shuttle protons from it. These contacts, in particular the ones with Tyr^318^ and Thr^320^, both in the β-hairpin loop, result in outer cavity opening. The orientations of both docked carbapenems expose their S-conjugate group to the solvent, with biapenem (Fig. 5a) exposed to a lesser extent than tebipenem (Fig. 5b).

## Discussion

Carbapenems inactivate transpeptidases by covalently binding to the enzyme’s catalytic serine or cysteine residue. Hydrolysis of this acyl-enzyme bond or transfer of the adducting acyl group to an acyl-acceptor reactivates the enzyme. Transpeptidases have evolved to transfer the acyl-adduct form in the first step of the transpeptidation to abundantly present in vivo acyl-acceptors, a process that is much faster than water-mediated hydrolysis of the acyl-enzyme (thio)ester bond. Hydrolysis of the thioester bond between these carbapenem and the catalytic cysteine is a very slow process in vitro [33–35]. Increased stability of the acyl-bond in a carbapenem adduct concerning acyl-transference is not expected, as this bond is not substantially different to that in the peptidyl-PG adduct. Whenever the acyl transfer step is not inhibited by the adduct, a rapid reactivation of the enzyme could be expected. Thus, carbapenems that form an adduct interfering with the acyl-transfer step may be better inactivators resulting in enhanced antimicrobial properties.

The stability of the enzyme-adduct bond to acyl-transfer has seldom been studied. Only very recently, Steiner *et al* [22] have shown that reactivation of Ldt_Mt2_-faropenem does not occur when *m*DAP is used as the acyl-acceptor, suggesting that the small β-OH-butyryl adduct remnant is enough to inhibit transpeptidation . However, reactivation by acyl-transfer still cannot be discarded; the same study observed an acyl-transfer-generated 6-aminopenicillanic acid dimer while studying Ldt_Mt2_ inactivation [22]. The single peptidyl stem residue *m*DAP binds to Ldt_Mt2_ weakly, as is suggested by the high concentration (2 mM) used to observe adducts, and larger PG fragments may overcome the adduct acyl-transfer inhibition.

**Fig. 6:**
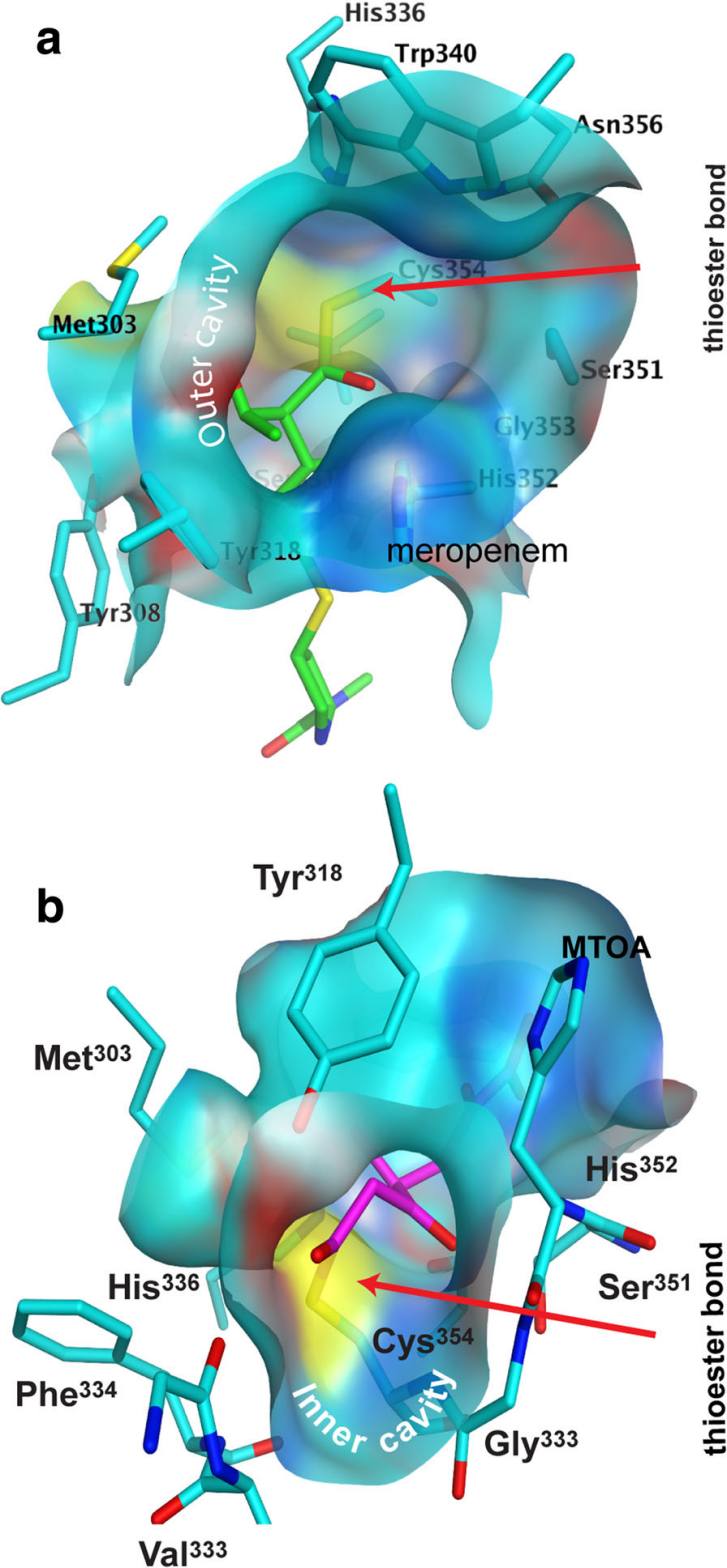
The different accessibilities of the thioester-bond in the two observed binding modes**. a** View from the outer cavity of the meropenem-adduct of Ldt_Mt2_ [20]. **b** View from the inner cavity of the MTOA-adduct of Ldt_Mt2_

The structures of carbapenems adducts of Ldt_Mt1_, Ldt_Mt2_, and Ldt_Mt5_ have shown that these carbapenems, despite being structurally similar, display two distinct modes of binding (Fig. 4b and d). Outer and inner cavity bound carbapenems create different access routes to the acyl-bond of the adduct by acyl-acceptors (Fig. 6), a factor that could affect negatively enzyme inactivation. Carbapenem adducts bound to the inner cavity of Ldts openly present the acyl-enzyme bond to an acyl-acceptor bound to the empty outer cavity (Fig. 6a). Instead, outer cavity adducts may inhibit acyl-acceptor recognition by Ldt motif residues that line the outer cavity (Fig. 6b). Even though previously proposed crosslinking mechanisms [13, 19] are in disagreement with the recognition of the acyl-donor substrate by Ldt_Mt2_, both agree the acyl-acceptor accesses the catalytic site from the outer cavity. Acyl-acceptors attacking the acyl-enzyme bond from the open inner cavity have poor specific recognition, potentially decreasing the chance of being optimally positioned for acyl-adduct transfer (Fig. 6b). Inner cavity adducts may have a reduced ability to prevent the reactivation of the enzyme by inhibiting acyl transfer to an acyl-acceptor coming from the outer cavity. Inner cavity adducts could disturb the outer cavity thereby impairing the recognition of acyl-acceptors (Fig. 4c), but there is a minimal effect on Ldt motif residues associated with acyl-acceptor recognition (Figs. 4c,d). Consequently, the inactivation of Ldts by outer-cavity forming adducts, like biapenem and tebipenem may be more effective as inhibitors in vivo.

Apparently, the carbapenems/Ldt_Mt2_ reactions display exothermic or endothermic enthalpic character depending on whether the adduct is bound to the Ldt_Mt2_ outer or inner cavity, respectively. Exothermic heat exchanges have been observed for the reaction with outer-cavity adduct forming carbapenems [21, 23]. In contrast, when Ldt_Mt2_ is titrated with the inner cavity adduct forming meropenem [18–20] an endothermic heat exchange has been observed [13]. With an expected similar free energy of acylation for both types of adducts, outer cavity bound adducts engage more polar residues—each providing a negative enthalpic contribution—than inner cavity bound adducts, which could explain the different enthalpic characteristics of the two binding modes.

The carbapenem adducts in both crystal forms studied here underwent loss of the C2 and C6 substituents. Comparing the observed adduct (Fig. 2b) to docking simulations of the intact carbapenems (Fig. 5), it is evident that the adduct degradation a) increases accessibility to the catalytic site of acyl-acceptors, and b) diminishes the inhibition of the acyl-acceptor recognition by increasing accessibility to the Ldt-motif residues that are responsible of acyl-acceptor recognition. Overall these modifications may affect the antibiotic potency by enabling the reactivation of the enzyme by the acyl-adduct transference to the abundant *in-vivo* acyl-acceptors. Thus, understanding the degradation mechanism is essential in rationally devising carbapenems resistant to degradation by the enzyme.

**Fig. 7:**
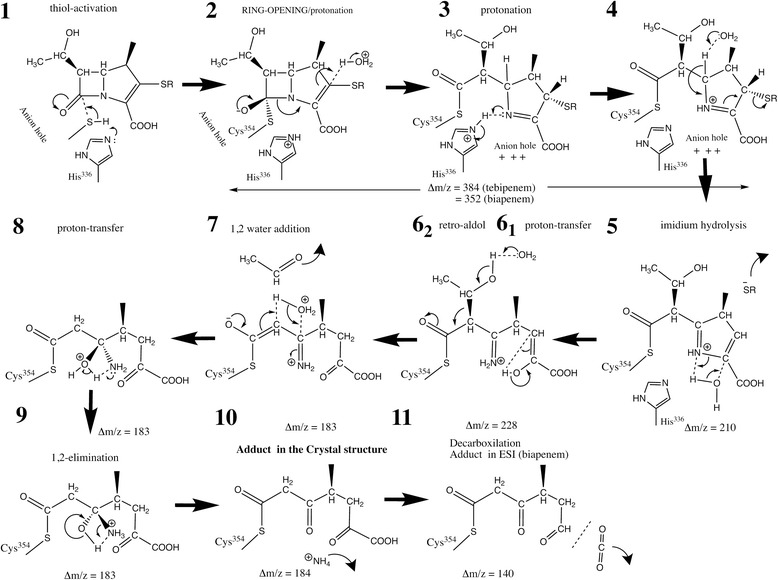
Proposed mechanisms of biapenem and tebipenem adduct formation and degradation. The figure shows the chemical steps (steps 1–10) to reach the crystallographically observed adducts from the expected initial (intact) carbapenem adduct of the enzyme. The expected mass differences in each step of the reaction are annotated for comparison with the observed species in the mass spectrometry experiments. A final decarboxylation step (step 11), where the product is observed in the ESI mass spectra but not in the crystal, is included as a possible explanation. The mass increases caused by the adducts were calculated using the program ChemDraw®(v 15.0.0.106; 1985-2015 PerkinElmer Informatics, Inc.)

We are proposing the mechanism of inactivation of Ldt_Mt2_ by biapenem and tebipenem and a possible path of degradation of these carbapenems on the basis of our crystallographic and mass spectrometry data (Fig. 7). As in the enzymatic mechanism proposed for serine and cysteine proteases, Ldt_Mt2_ residue His^336^ is poised to deprotonate Cys^354^, converting it to a nucleophilic thiolate that attacks the carbonyl carbon at position 7 (Fig. 1d) of the carbapenem (Fig. 7, step 1). Residues in the loop containing the catalytic cysteine (residues 352–354) form the oxyanion hole by having their main chain NH groups oriented so as to concentrate their residual positive charge toward a pocket. In this pocket, the oxygen of the carbonyl group at C7 forms hydrogen bonds to His^352^ and Cys^354^ main chain atoms (Fig. 5). The acylation is aided by the oxyanion-hole stabilization of the negative charge formed at the oxygen in the tetrahedral intermediate (Fig. 7, step 2). The acylation causes β-lactam ring opening and the formation of an imine, while the pyrroline ring tautomerizes to a more stable Δ^1^ tautomer by acquiring a proton at C2 (Fig. 7, step 2). Up to here, the reaction produces the acyl-enzyme product expected when a β-lactam compound reacts with a thiolate. However, the observed electron density suggests the loss of the group at C2 and opening of pyrroline ring. Thus, we are proposing the following degradation mechanism. Following acylation, the N4 group of the open carbapenem is in close proximity to the charged His^336^ and over the oxyanion hole, positioning it to withdraw the proton from His^336^ previously abstracted from Cys^354^ (Fig. 7, step 3). The net positive charge of the resulting iminium and oxyanion hole increases the proton acidity at C5 facilitating its abstraction by a base required to trigger the following steps. After the proton abstraction, the resulting compound undergoes thiol-conjugate elimination (Fig. 7, step 4), followed by the opening of the ring by a nucleophilic attack by water (Fig. 7, step 5). A proton-transfer tautomerizes the enol form of the pyruvate group to a more stable keto form (Fig. 7, step 6_1_). The mass of the resulting compound (Δm/z = 228 Da) is in good agreement with the difference between primary signals observed by MALDI-TOF of the Ldt_Mt2_ biapenem reaction after a short incubation (Δm/z = 225 Da, Table 3). The mechanism described in steps 4–5 is similar to the last step of the thiol-conjugated group elimination by β-lyases [36]. These enzymes catalyze the reaction RSCHCH(COO^−^)NH_3_^+^ + H_2_O → RSH + NH_4_^+^ + pyruvate [37].

Although both complex crystals showed the same adduct lacking the thiol-conjugate substituent, the mass change observed in tebipenem samples following a 24 h incubation are larger than expected (Tables 2 and 3). The elimination 1-(4,5-dihydrothiazol-2-yl)azetidine-3-thiol substituent of the tebipenem adduct is observed in the crystal (which formed over the course of many days) suggesting that this elimination is a much slower process than the rapid elimination of the (bicyclotriazolium)-thio group of the biapenem adduct (<15 min, Table 3). The substituent at C2 is likely eliminated from the core of the different carbapenems at different rates. In β-lyases [36] electron-withdrawing groups conjugated to the thiol, such as the bicyclotriazolium group in biapenem, undergo more rapid elimination. The elimination of the thiolsulfide group at C2 results in a positively charged iminium substituent at C5 (Fig. 7, step 7). The docking of this substituent at the oxyanion hole and its close coordination by a high number of hydrogen-bond donors (main-chain of Cys^354^, Gly^353^, and His^352^) observed in the crystal structure (Fig. 2a) suggests the non proton-acceptor iminium group is hydrolyzed to a ketone (Fig. 7, steps 7–10).

The electron density of the hydroxyethyl substituent at C6 was absent in both crystal forms, suggesting the elimination of this group as acetaldehyde by a retro-aldol reaction (Fig. 7, step 6_2_). The 45 Da decrease in the mass in both adducts detected by mass spectrometry experiments (Table 2 and 3) are compatible with the proposed elimination (Fig. 7, step 6_2_). Decarboxylation (expected Δm/z = -44 Da) is also a possibility although it is not supported by our crystallographic results. The elimination of this group *via* a retro-aldol reaction has also been proposed to occur in class A and C β-lactamases adducts [12, 38, 39]. The hydroxyethyl substituent elimination occurred at substantially different rates for each carbapenem. In the tebipenem sample, this occurred before elimination of the C2 substituent was detectable, while in biapenem it occurred immediately and apparently before C2 substituent elimination (Tables 2 and 3). After a 45 min incubation of biapenem and Ldt_Mt2_, the difference between mass spectra primary signals stabilized at around a 180 Da, which is 45 Da less than difference observed after a short incubation (Table 3) and closely matches the difference expected (183 Da, Fig. 7, step 7).

Not all carbapenems with a thiol conjugate group at C2 are expected to degrade via the pathway outlined in Fig. 7. Factors such as the electron withdrawing properties of the thiol substituent and/or binding site interactions with substituent groups could control the degradation. For example, in the Ldt_Mt1_-imipenem adduct the hydroxyethyl substituent is not eliminated, perhaps because this group is better accommodated in the Ldt_Mt1_ binding site due to the displaced position of the β-hairpin loop (Fig. 4b). The loss of the hydroxyethyl substituent of biapenem and tebipenem allows the Ldt_Mt2_-adduct to adopt a closed conformation of the β-hairpin loop (Fig. 1a). Conversely, the open conformation is observed in the meropenem adduct [19] in which that group interacts with Tyr^318^ at the β-hairpin loop (Fig. 4). Mutations of Tyr^318^ to either phenylalanine or alanine have been shown to enhance meropenem hydrolysis [20]. These mutations remove contacts between adduct and β-hairpin loop that may restore active conformations of the enzyme.

Decarboxylation of the MTOA adduct results in a Δm/z of 140.1 Da, that closely matches the 139.5 Da observed for Ldt_Mt2_-biapenem by mass spectrometry (Table 2). The decarboxylation may result from the sampling conditions (exposure to trifluoroacetic acid, temperature 60 °C, etc.). Few biapenem degradation paths exist yielding this observed adduct mass. Thus, this finding provides strong additional evidence supporting the identification of MTOA as the adduct observed in the Ldt_Mt2_-biapenem crystal.

## Conclusions

In summary, biapenem and tebipenem bind to the outer cavity of Ldt_Mt2_ and inactivate the enzyme, ultimately forming the same adduct following degradation. These new Ldt-carbapenem structures provide additional evidence that binding to the outer cavity is the predominant mode by which carbapenems bind to *Mtb* Ldts. Although the reaction enthalpy data are limited to a small number of inner-cavity bound carbapenems, the apparent correlation between binding mode and thermodynamic trend suggests a simple method to determine the binding mode: exothermic reaction profiles are indicative of binding to the outer cavity, while endothermic reaction profiles may indicate binding to the inner cavity.

Carbapenems that form outer-cavity adducts have the potential to be better inhibitors than inner-cavity adducts, as they may engage Ldt motif residues that sequentially recognize acyl-donor and acyl-acceptor PG stem peptide substrates. This and other recent studies [21, 22] show that outer-cavity adducts are subject to Ldt_Mt2_ catalyzed degradation that may reduce their interference with in vivo acyl-transfer, highlighting the need to consider binding mode and resistance to enzymatic degradation in antibiotic design. Kinetic studies examining the difference between enzymatic rates of acylation and deacylation by hydrolysis or acyl group transference to in vivo substrates are warranted, as the resulting rate of inactivation will assist in the rational design of antibiotics.

Importantly, the observed elimination of thiol-conjugate group at C2 could be devised as a means of targeted and localized release of a synergistic antimicrobial factor carried by a carbapenem in the form of an S-conjugated substituent. As attempted in cephalosporin-based biofilm dispersing NO-donor pro-drugs activated by β-lactamases [40], the design of Ldt activated pro-drugs based on the carbapenem scaffold might also be worth pursuing.

## Additional file

Additional file 1: Text. Collision induced fragmentation of AP-MALDI molecular ion analysis. **Figure S1.** Views of the electron density associated with the active site and refined models. **Figure S2.** Atmosphere pressure MALDI analysis of carbapenem reagent used. **Figure S3.** PLC-HRMS (ESI) of Inhibited Ldt_Mt2_ species. **Figure S4.** MALDI TOF of the reaction time course. (PDF 895 kb)

## Abbreviations

AP-MALDI: Atmospheric pressure matrix-assisted laser desorption/ionization
BIg: Bacterial Ig-like domain
CD: Catalytic domain
ESI-TOF MS: Electrospray ionization time of flight mass spectrometry
Ldt: L,D-transpeptidase
MALDI-TOF: Matrix-assisted laser desorption/ionization time of flight mass spectrometry
*m*DAP: *meso*-diaminopimelic acid
*Mtb*: *Mycobacterium tuberculosis*
MTOA: (*S*)-4-methyl-2,5,7-trioxoheptanoic acid
PG: Peptidoglycans

## References

[1] Walsh C (2003). Antibiotics: Actions, Origins, Resistance.

[2] Cho H, Uehara T, Bernhardt TG (2014). β-lactam antibiotics induce a lethal malfunctioning of the bacterial cell wall synthesis machinery. Cell.

[3] Hamad B (2010). The antibiotics market. Nat Rev Drug Discov.

[4] WHO (2015). Global Tuberculosis Report.

[5] Hugonnet JE, Blanchard JS (2007). Irreversible inhibition of the *Mycobacterium tuberculosis* β-lactamase by clavulanate. Biochemistry.

[6] Wivagg CN, Bhattacharyya RP, Hung DT (2014). Mechanisms of β-lactam killing and resistance in the context of *Mycobacterium tuberculosis*. J Antibiotics.

[7] Mainardi JL, Fourgeaud M, Hugonnet JE, Dubost L, Brouard JP, Ouazzani J, Rice LB, Gutmann L, Arthur M (2005). A novel peptidoglycan cross-linking enzyme for a β-lactam-resistant transpeptidation pathway. J Biol Chem.

[8] Wietzerbin J, Das BC, Petit JF, Lederer E, Leyh-Bouille M, Ghuysen JM (1974). Occurrence of D-alanyl-(D)-meso-diaminopimelic acid and meso-diaminopimelyl-meso-diaminopimelic acid interpeptide linkages in the peptidoglycan of Mycobacteria. Biochemistry.

[9] Lavollay M, Arthur M, Fourgeaud M, Dubost L, Marie A, Veziris N, Blanot D, Gutmann L, Mainardi JL (2008). The peptidoglycan of stationary-phase *Mycobacterium tuberculosis* predominantly contains cross-links generated by L,D-transpeptidation. J Bacteriol.

[10] Gupta R, Lavollay M, Mainardi JL, Arthur M, Bishai WR, Lamichhane G (2010). The *Mycobacterium tuberculosis* protein Ldt_Mt2_ is a nonclassical transpeptidase required for virulence and resistance to amoxicillin. Nat Med.

[11] Kumar P, Arora K, Lloyd JR, Lee IY, Nair V, Fischer E, Boshoff HI, Barry CE (2012). Meropenem inhibits D, D-carboxypeptidase activity in Mycobacterium tuberculosis. Mol Microbiol.

[12] Hugonnet JE, Tremblay LW, Boshoff HI, Barry CE, Blanchard JS (2009). Meropenem-clavulanate is effective against extensively drug-resistant *Mycobacterium tuberculosis*. Science (New York NY).

[13] Erdemli SB, Gupta R, Bishai WR, Lamichhane G, Amzel LM, Bianchet MA (2012). Targeting the cell wall of Mycobacterium tuberculosis: structure and mechanism of L, D-transpeptidase 2. Structure.

[14] Dhar N, Dubee V, Ballell L, Cuinet G, Hugonnet JE, Signorino-Gelo F, Barros D, Arthur M, McKinney JD (2015). Rapid Cytolysis of *Mycobacterium tuberculosis* by Faropenem, an Orally Bioavailable β-Lactam Antibiotic. Antimicrob Agents Chemother.

[15] Brammer Basta LA, Ghosh A, Pan Y, Jakoncic J, Lloyd EP, Townsend CA, Lamichhane G, Bianchet MA (2015). Loss of a Functionally and Structurally Distinct L, D-Transpeptidase, LdtMt5, Compromises Cell Wall Integrity in Mycobacterium tuberculosis. J Biol Chem.

[16] Kieser KJ, Baranowski C, Chao MC, Long JE, Sassetti CM, Waldor MK, Sacchettini JC, Ioerger TR, Rubin EJ (2015). Peptidoglycan synthesis in *Mycobacterium tuberculosis* is organized into networks with varying drug susceptibility. Proc Natl Acad Sci U S A.

[17] Schoonmaker MK, Bishai WR, Lamichhane G (2014). Nonclassical transpeptidases of *Mycobacterium tuberculosis* alter cell size, morphology, the cytosolic matrix, protein localization, virulence, and resistance to β-lactams. J Bacteriol.

[18] Both D, Steiner EM, Stadler D, Lindqvist Y, Schnell R, Schneider G (2013). Structure of LdtMt2, an L, D-transpeptidase from Mycobacterium tuberculosis. Acta Crystallogr Sect D: Biol Crystallogr.

[19] Kim HS, Kim J, Im HN, Yoon JY, An DR, Yoon HJ, Kim JY, Min HK, Kim SJ, Lee JY (2013). Structural basis for the inhibition of Mycobacterium tuberculosis L, D-transpeptidase by meropenem, a drug effective against extensively drug-resistant strains. Acta Crystallogr Sect D: Biol Crystallogr.

[20] Li WJ, Li DF, Hu YL, Zhang XE, Bi LJ, Wang DC (2013). Crystal structure of L, D-transpeptidase LdtMt2 in complex with meropenem reveals the mechanism of carbapenem against Mycobacterium tuberculosis. Cell Res.

[21] Kumar P, Kaushik A, Lloyd EP, Li SG, Mattoo R, Ammerman NC, Bell DT, Perryman AL, Zandi TA, Ekins S (2017). Non-classical transpeptidases yield insight into new antibacterials. Nat Chem Biol.

[22] Steiner EM, Schneider G, Schnell R. Binding and processing of β-lactam antibiotics by the transpeptidase Ldt_Mt2_ from *Mycobacterium tuberculosis*. FEBS J. 2017. 10.1111/febs.14010 28075068

[23] Correale S, Ruggiero A, Capparelli R, Pedone E, Berisio R (2013). Structures of free and inhibited forms of the L, D-transpeptidase LdtMt1 from Mycobacterium tuberculosis. Acta Crystallogr Sect D: Biol Crystallogr.

[24] Horita Y, Maeda S, Kazumi Y, Doi N (2014). In vitro susceptibility of *Mycobacterium tuberculosis* isolates to an oral carbapenem alone or in combination with β-lactamase inhibitors. Antimicrob Agents Chemother.

[25] Kaushik A, Makkar N, Pandey P, Parrish N, Singh U, Lamichhane G (2015). Carbapenems and Rifampin Exhibit Synergy against *Mycobacterium tuberculosis* and *Mycobacterium abscessus*. Antimicrob Agents Chemother.

[26] Pettersen EF, Goddard TD, Huang CC, Couch GS, Greenblatt DM, Meng EC, Ferrin TE (2004). UCSF Chimera--a visualization system for exploratory research and analysis. J Comput Chem.

[27] Gill SC, von Hippel PH (1989). Calculation of protein extinction coefficients from amino acid sequence data. Anal Biochem.

[28] Otwinowski Z, Minor W (1997). Processing of X-ray diffraction data collected in oscillation mode. Methods Enzymol.

[29] McCoy AJ (2007). Solving structures of protein complexes by molecular replacement with Phaser. Acta Crystallogr Sect D: Biol Crystallogr.

[30] Adams PD, Afonine PV, Bunkoczi G, Chen VB, Davis IW, Echols N, Headd JJ, Hung LW, Kapral GJ, Grosse-Kunstleve RW (2010). PHENIX: a comprehensive Python-based system for macromolecular structure solution. Acta Crystallogr Sect D: Biol Crystallogr.

[31] Emsley P, Lohkamp B, Scott WG, Cowtan K (2010). Features and development of Coot. Acta Crystallogr Sect D: Biol Crystallogr.

[32] Schmidt MW, Baldrige KK, Boatz JA, Elbert ST, Gordon MS, Jensen JH, Koseki S, Matsunaga N, Nguyen KA, Su SJ (1993). General Atomic and molecular Electronic-Structure System. J Comput Chem.

[33] Dubee V, Arthur M, Fief H, Triboulet S, Mainardi JL, Gutmann L, Sollogoub M, Rice LB, Etheve-Quelquejeu M, Hugonnet JE (2012). Kinetic analysis of Enterococcus faecium L, D-transpeptidase inactivation by carbapenems. Antimicrob Agents Chemother.

[34] Dubee V, Triboulet S, Mainardi JL, Etheve-Quelquejeu M, Gutmann L, Marie A, Dubost L, Hugonnet JE, Arthur M (2012). Inactivation of Mycobacterium tuberculosis L, D-transpeptidase LdtMt1 by carbapenems and cephalosporins. Antimicrob Agents Chemother.

[35] Triboulet S, Dubee V, Lecoq L, Bougault C, Mainardi JL, Rice LB, Etheve-Quelquejeu M, Gutmann L, Marie A, Dubost L (2013). Kinetic features of L, D-transpeptidase inactivation critical for β-lactam antibacterial activity. PLoS One.

[36] Eliot AC, Kirsch JF (2004). Pyridoxal phosphate enzymes: mechanistic, structural, and evolutionary considerations. Annu Rev Biochem.

[37] Cooper AJ (1998). Mechanisms of cysteine S-conjugate β-lyases. Adv Enzymol Relat Areas Mol Biol.

[38] Drawz SM, Babic M, Bethel CR, Taracila M, Distler AM, Ori C, Caselli E, Prati F, Bonomo RA (2010). Inhibition of the class C β-lactamase from Acinetobacter spp.: insights into effective inhibitor design. Biochemistry.

[39] Endimiani A, Luzzaro F, Pini B, Amicosante G, Rossolini GM, Toniolo AQ (2006). *Pseudomonas aeruginosa* bloodstream infections: risk factors and treatment outcome related to expression of the PER-1 extended-spectrum β-lactamase. BMC Infect Dis.

[40] Yepuri NR, Barraud N, Mohammadi NS, Kardak BG, Kjelleberg S, Rice SA, Kelso MJ (2013). Synthesis of cephalosporin-3′-diazeniumdiolates: biofilm dispersing NO-donor prodrugs activated by β-lactamase. Chem Commun (Camb).

