# Supplementary material for: Structural insight into the inactivation of Mycobacterium tuberculosis non-classical transpeptidase LdtMt2 by biapenem and tebipenem
Source: BMC Biochem. 2017 May 25;18:8. doi: 10.1186/s12858-017-0082-4 (PMC5445500; doi:10.1186/s12858-017-0082-4)
Supplement: Additional file 1: — Text. Collision induced fragmentation of AP-MALDI molecular ion analysis. Figure S1. Views of the electron density associated with the active site and refined models. Figure S2. Atmosphere pressure MALDI analysis of carbapenem reagent used. Figure S3. PLC-HRMS (ESI) of Inhibited LdtMt2 species. Figure S4. MALDI TOF of the reaction time course. (PDF 895 kb) [file 12858_2017_82_MOESM1_ESM.pdf]

Supplementary material

Structural insight into the inactivation of *Mycobacterium tuberculosis* non-classical transpeptidase by biapenem and tebipenem

Mario A. Bianchet[1,6,♯,*], Ying Pan[1♯], Leighanne A. Brammer Basta[2], Harry Saavedra[1], Evan P. Lloyd[3], Pankaj Kumar[4], Rohini Mattoo[4], Craig A. Townsend[3,5], and Gyanu Lamichhane[4,5]*

[1]Structural Enzymology and Thermodynamics Group, Department of Biophysics and Biophysical Chemistry, and [6]Department of Neurology, Johns Hopkins University School of Medicine, Baltimore, MD 21205

[2]Chemistry Department, United States Naval Academy, Annapolis, MD 21402

[3]Department of Chemistry, Johns Hopkins University, Baltimore, MD 21218

[4]Center for Tuberculosis Research, Johns Hopkins University, and [5]Taskforce to study Resistance Emergence & Antimicrobial development Technology (TREAT), Division of Infectious Diseases, Johns Hopkins University, Baltimore, MD 21231

*To whom correspondence should be addressed: Gyanu Lamichhane, Johns Hopkins University School of Medicine, 1503 E. Jefferson Street, Baltimore, MD 21231, USA; Tel.: (410)502-8162; E-mail: lamichhane@jhu.edu or Mario A Bianchet, Departments of Neurology and Biophysics and Biophysical Chemistry, Johns Hopkins University School of Medicine, 725 N. Wolfe Street, Baltimore, MD 21205, USA; Tel.: (410)614-8221; E-mail: bianchet@jhmi.edu

[♯]These authors have equally contributed to this work

Supplementary Text

Collision induced fragmentation of AP-MALDI molecular ion analysis

For both compounds, the molecular ion ($[M+H]^+$) was isolated in the ion trap and collided with Argon gas of certain energy (MS/MS). The $[M-86+H]^+$ species —m/z 265.08 and 298.08 Da for biapenem and tebipenem, respectively— were by far the major detected fragment from collision-induced decomposition (**Fig. S2d-e)**. These fragments are facile retro [2+2] cleavage of the β-lactam ring of the carbapenems. At low collision energies, the relative intensities of $[M-86+H]^+$ species were similar for both compounds, indicating that the structurally homologous biapenem and tebipenem carbapenems share a common fragmentation pathway that has the lowest energy threshold for collision-induced fragmentation. However, in the AP-MALDI experiments, this fragment has similar signal intensity for the tebipenem sample, but is not detected in the biapenem sample (**Fig. S2b-c**). Therefore, we can conclude that the $[M-86+H]^+$ species detected in AP-MALDI of the tebipenem sample may be not a result of ligand fragmentation during the AP-MALDI experiment, but an impurity already present in the sample itself. None of the impurities detected correspond to any of the masses observed in the crystal forms.

Figure Captions

Figure S1 | Views of the electron density associated with the active site and refined models. Simulated annealing omit map density corresponding to the observed adduct. (**a**) Stereo view from the inner cavity of the Ldt_{Mt2}-biapenem crystal, the electron density map (blue) omitting the MTOA (magenta) was calculated using the program Phenix after a refinement cycle including torsional simulated annealing step with MTOA omitted (occupancy 0.0). (**b**) Stereo view from the outer cavity of the Ldt_{Mt2}-tebipenem, omit electron density is shown colored in orange. The omit maps were contoured a 3.5 σ. (**c**) Stereo view of the refined density at the binding site of the apo-Ldt_{MT2}. The observed glycerol molecule (magenta) and 2DFo-mFc sigmaA-weighted electron density map (blue) contoured at 1 σ at the outer cavity site are shown. (**d**) The accessible surface of the inner cavity showing the electron density (2mFo-DFc map colored in pink and contoured at 0.9 sigmas) around the hydroxyethyl remnant (orange) the MTOA and residues in the active site (cyan). (**e**) Overlay of the catalytic site of apo-Ldt_{Mt2} (green) and Ldt_{Mt2}-biapenem adduct crystal structures (cyan). Only one atom of MTOA adduct (cyan) overlap with other of the glycerol molecule (green). The distance from the glycerol nearest atom to Cys^{354} sulfur atom is marked with a blue dashed line line.

Figure S2 | **Atmosphere pressure MALDI analysis of carbapenem reagent used** (**a**) 10 µM biapenem AP-MALDI, (**b**) 13 µM, tebipenem AP-MALDI, (**c**) Matrix CHOCH 1 mg/ml, (**d**) MS/MS of molecular ion of biapenem and **(e)** MS/MS of molecular ion of tebipenem.

Figure S3 | UPLC-HRMS (ESI) of Inhibited Ldt_{MT2} species Intact protein masses as calculated from m/z data for:(**a_1**) – native Ldt_{MT2}, (**b_1**) – Ldt_{Mt2} inhibited with biapenem, (**c_1**) – Ldt_{Mt2} inhibited with tebipenem. The greyed area is the range of masses examined in processing. (**a_2**), (**b_2**) and (**c2**) represent the total ion chromatograms of the injected samples examined in **a**, **b** and **c** respectively. The greyed area is the selected window of retention for data processing.

Figure S4 | MALDI TOF of the reaction time course (**a**) Apo Ldt_{Mt2}, (**b**) after reacting with biapenem for 15 min and (**c**) after 40 min at RT. Mass spectra after reacting with tebipenem for (**d**) 40 min at RT and (**e**) after 24 h at 4 ^{o}C. A larger m/z observed for the apo-Ldt_{Mt2} (38198 Da) than the mass/z expected (38085 Da) is caused for the formation of Na adducts (100 mM of NaCl is used in the reaction buffer for protein stability). The theoretical average mass including amino acids from the TEV cleavage site and linker of 38085 Da was calculated using the ExPASy mass calculator server (http://web.expasy.org/compute_pi/).

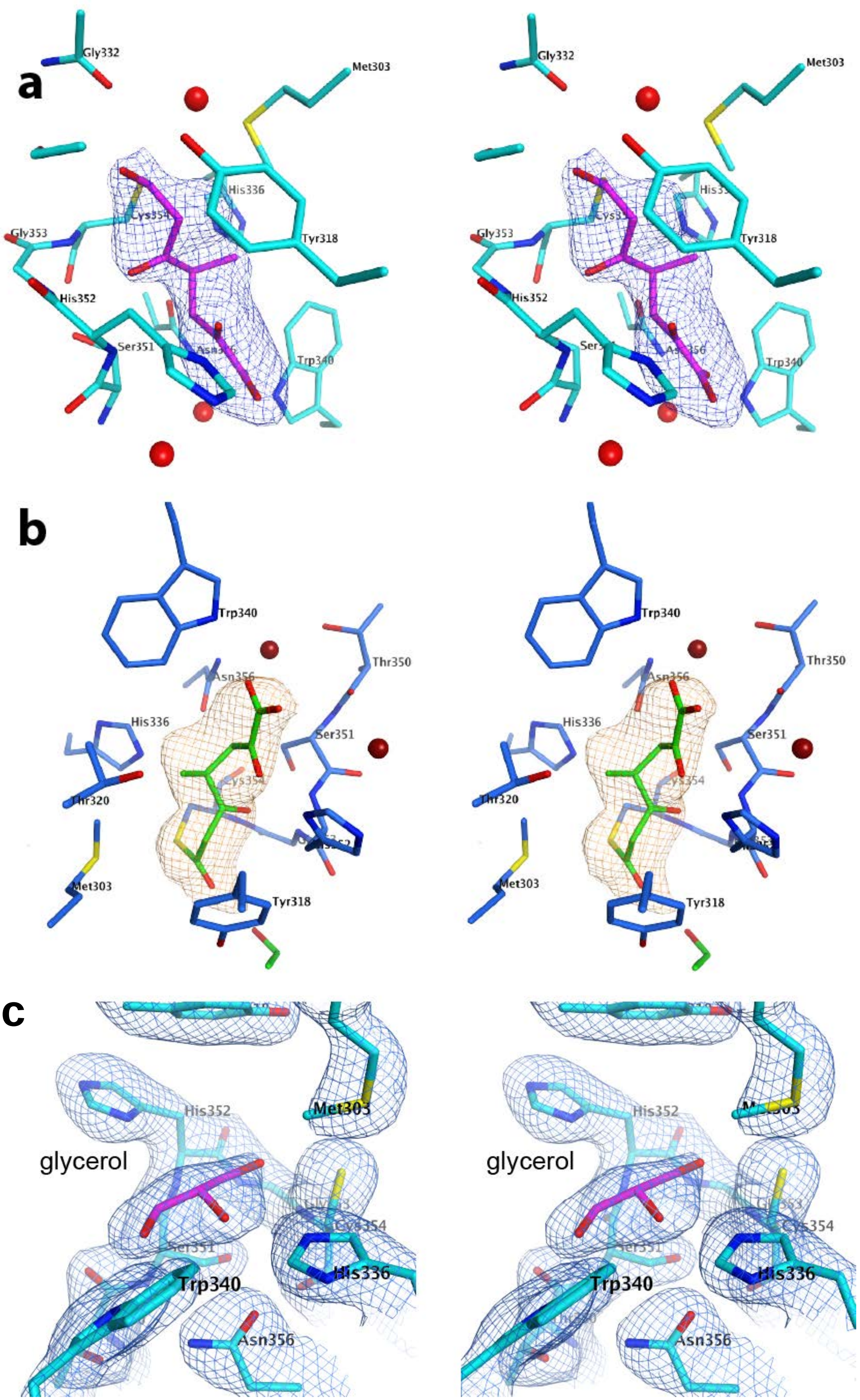

Figure S1a, b, and c

Figure S1d

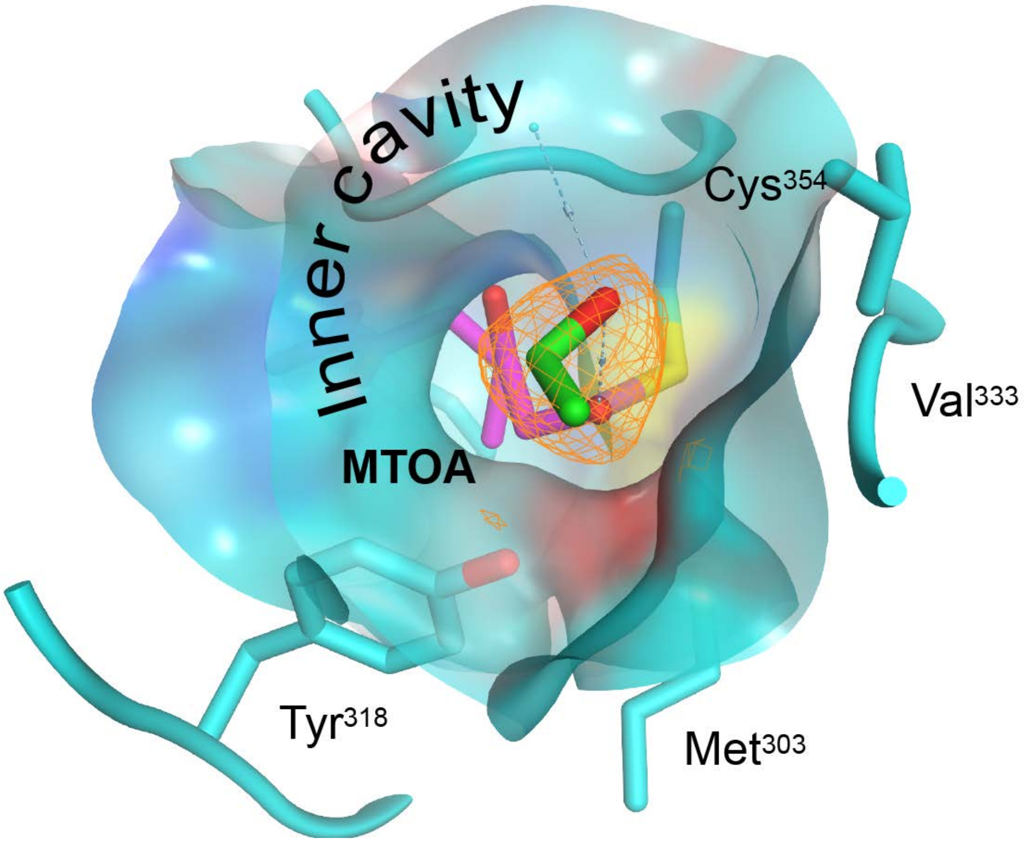

Figure S1e

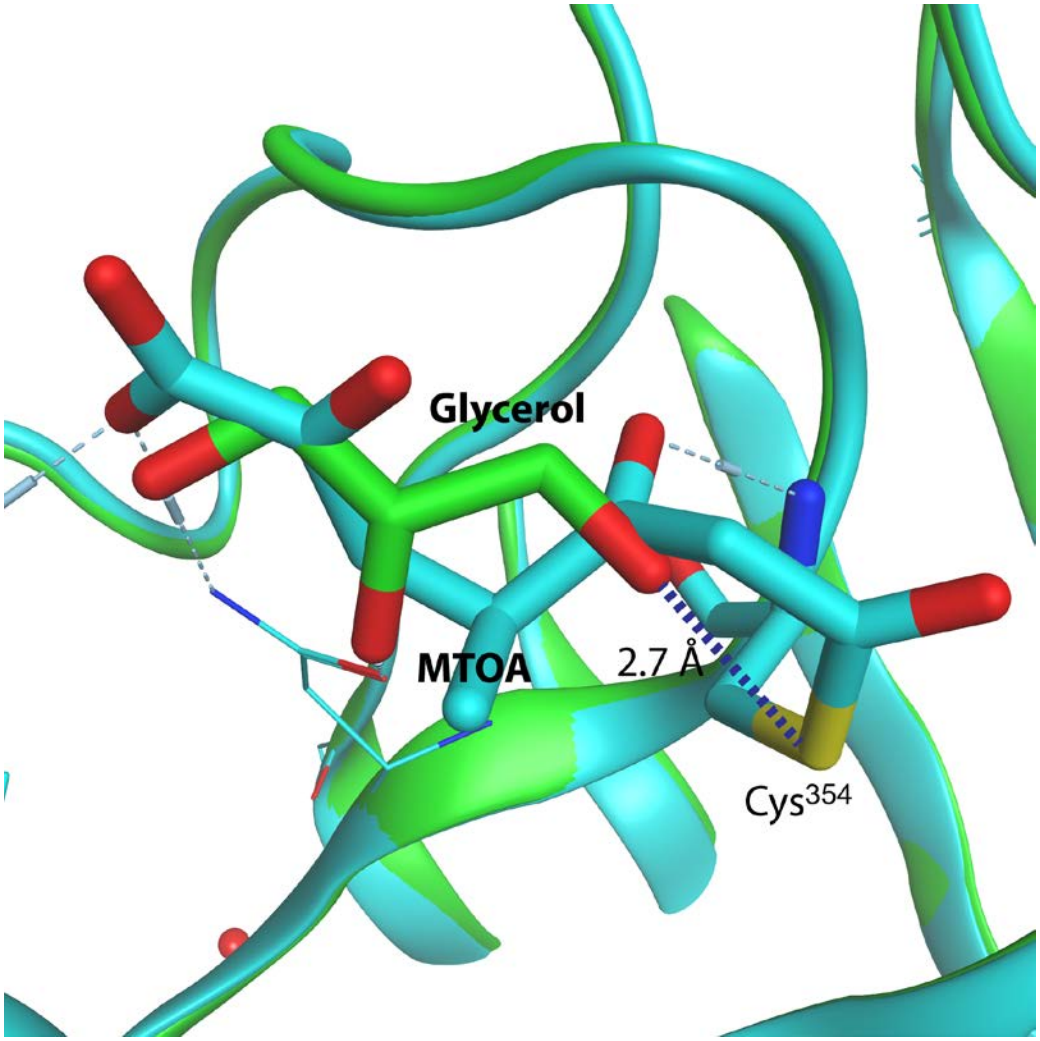

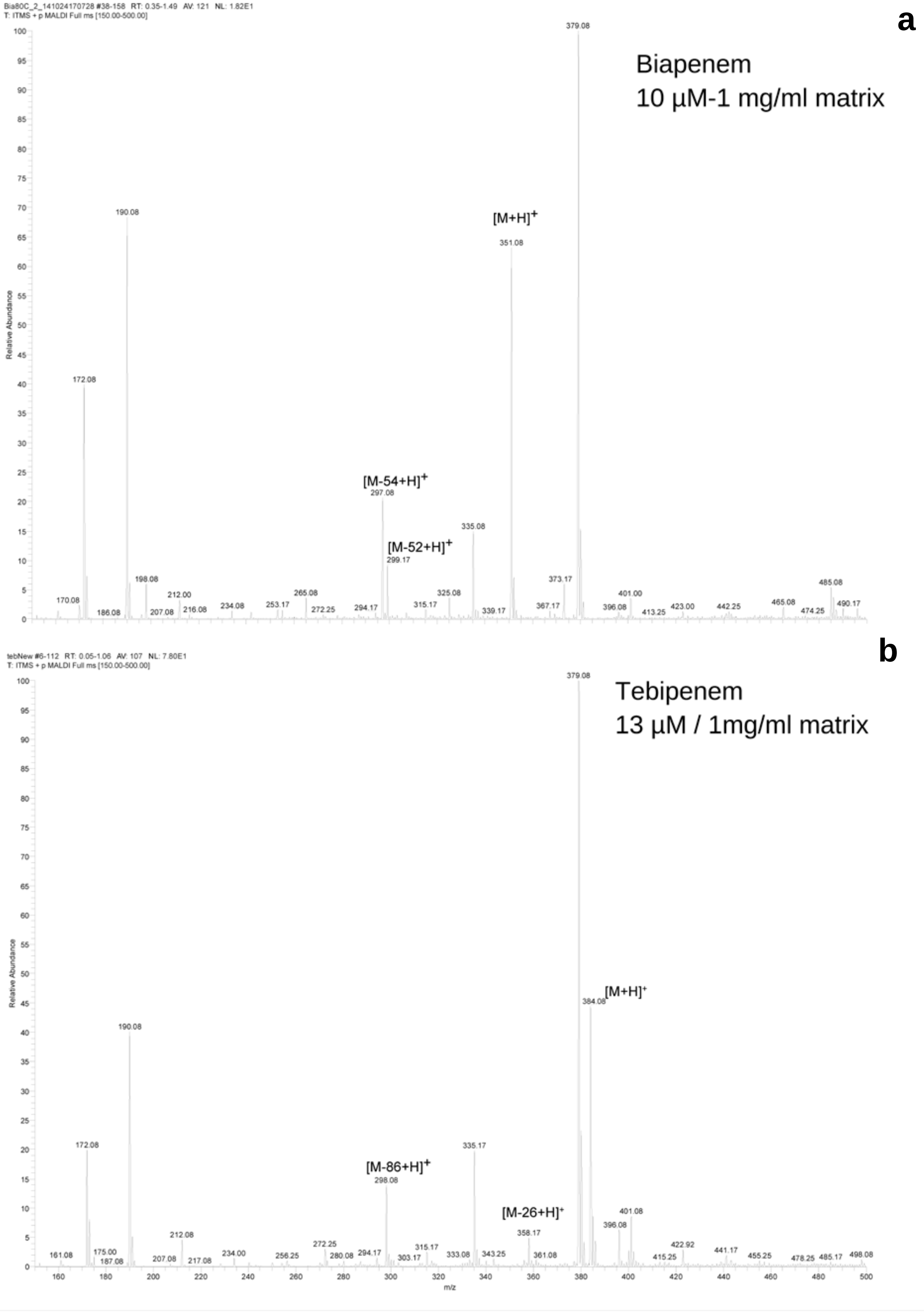
a
Biapenem
10 µM-1 mg/ml matrix
[M+H]+
351.08
379.08
190.08
172.08
[M-54+H]+
297.08
[M-52+H]+
299.17
335.08
Relative Abundance
b
Tebipenem
13 µM / 1mg/ml matrix
[M+H]+
384.08
379.08
190.08
172.08
[M-86+H]+
298.08
335.17
[M-26+H]+
358.17
Relative Abundance
m/z

Figure S2a,b

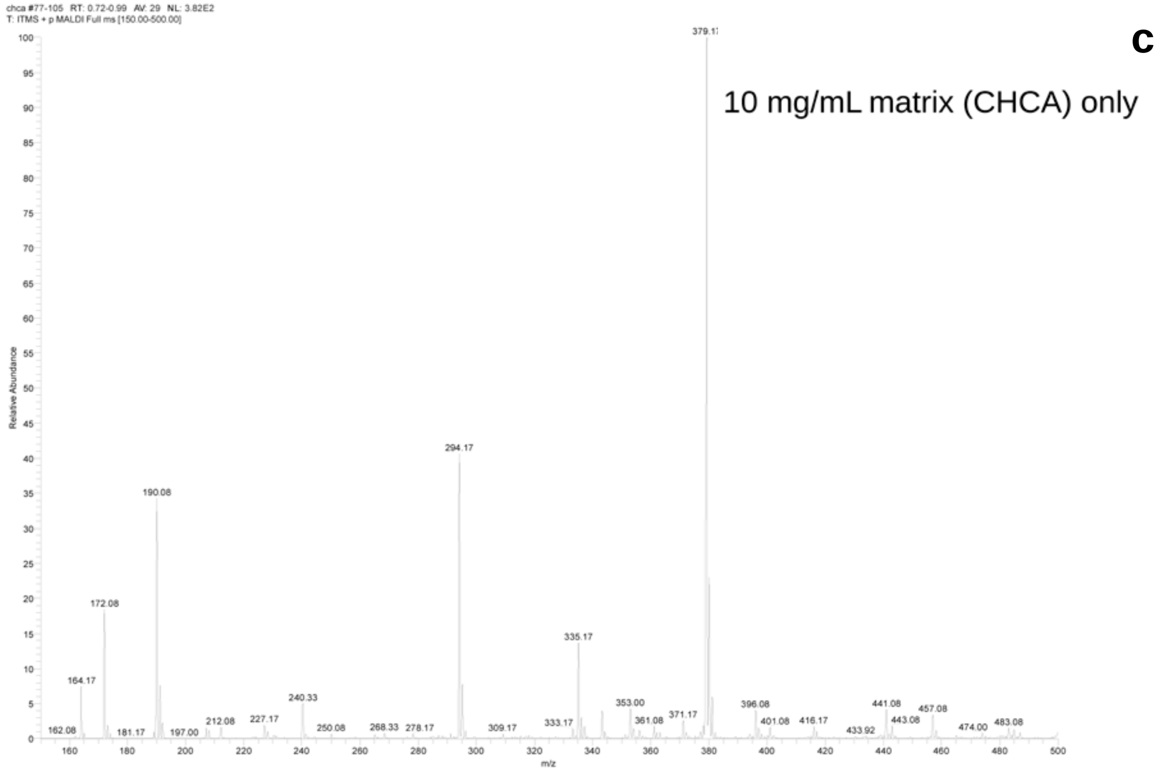
c
10 mg/mL matrix (CHCA) only
chca #77-105 RT: 0.72-0.99 AV: 29 NL: 3.82E2
T: ITMS + p MALDI Full ms [150.00-500.00]
Relative Abundance
m/z
379.17
294.17
190.08
172.08
164.17
335.17
240.33
353.00
396.08
441.08
457.08

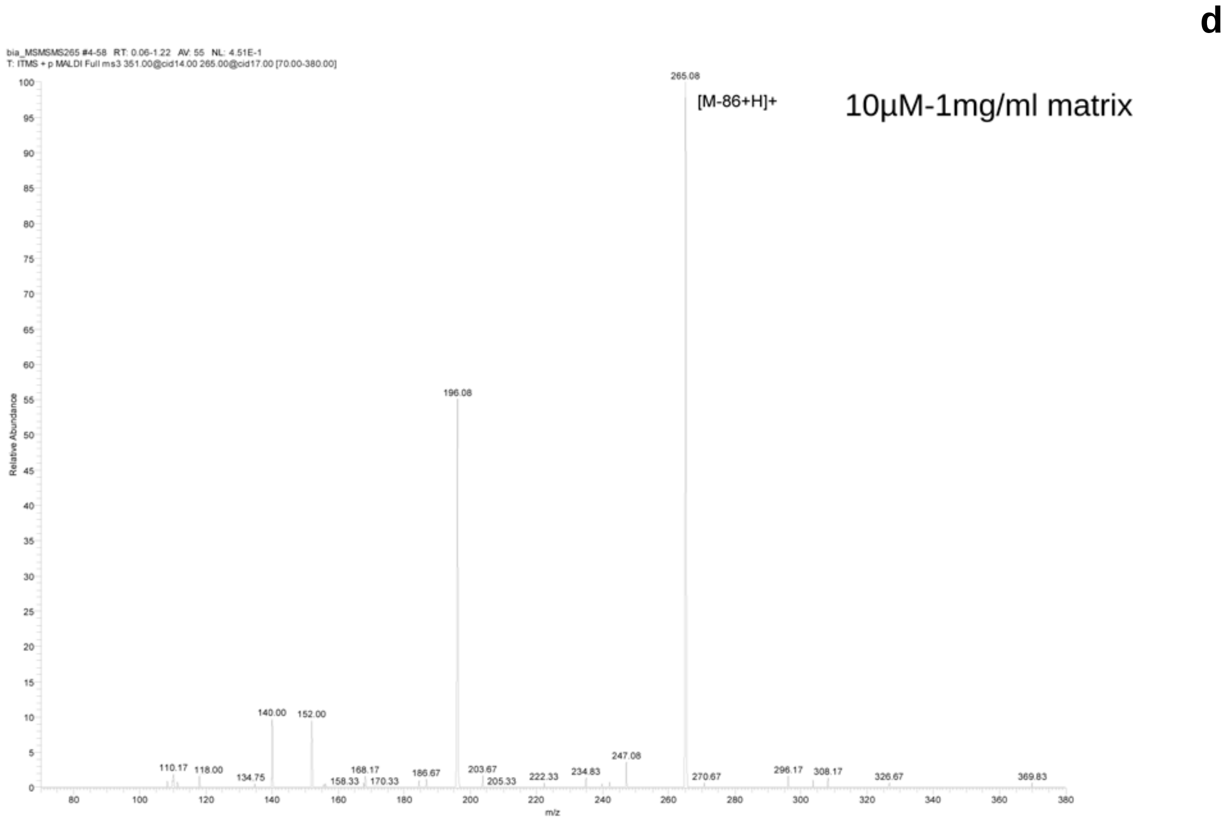
d
10µM-1mg/ml matrix
[M-86+H]+
bia_MSMSMS265 #4-58 RT: 0.06-1.22 AV: 55 NL: 4.51E-1
T: ITMS + p MALDI Full ms3 351.00@cid14.00 265.00@cid17.00 [70.00-380.00]
Relative Abundance
m/z
265.08
196.08
140.00
152.00
247.08

Figure S2c,d

e

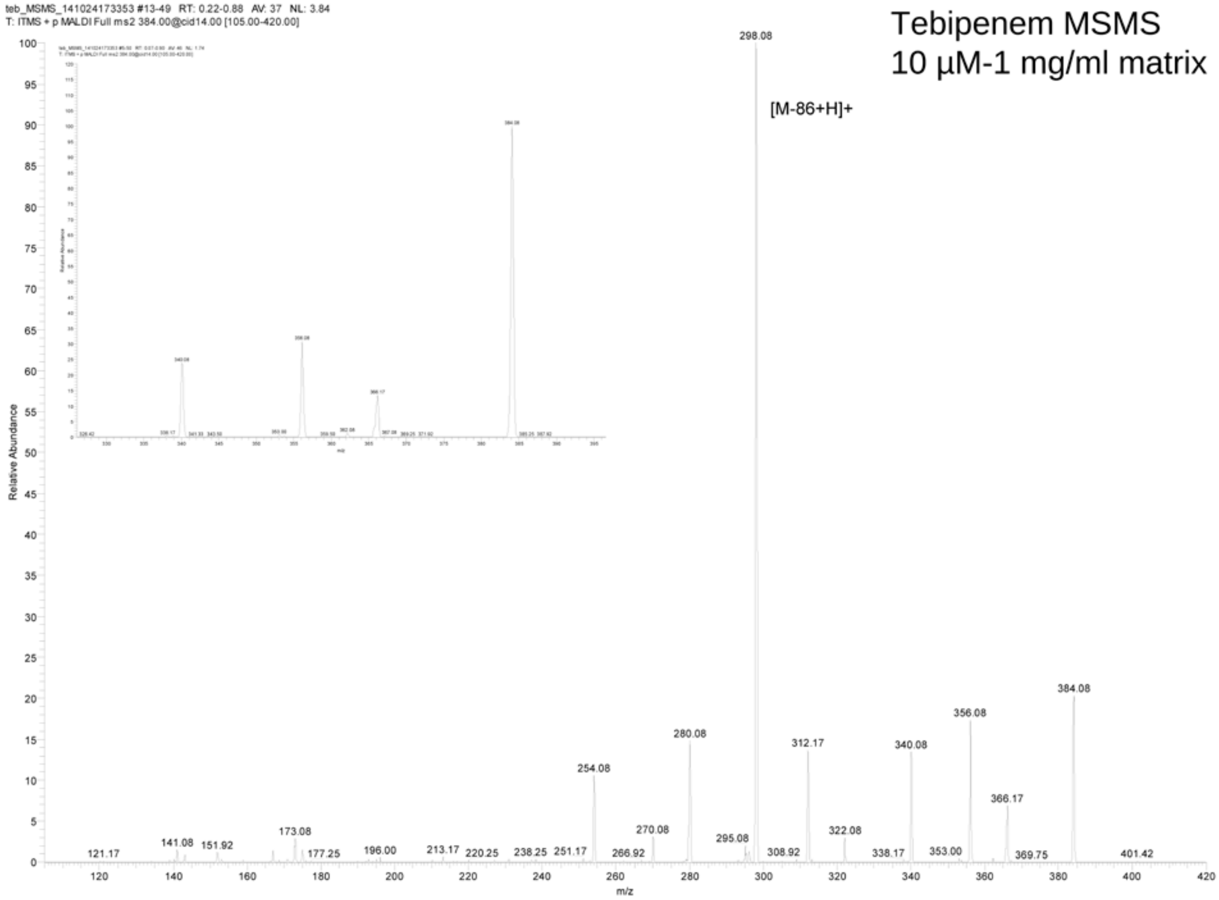

teb_MSMS_141024173353 #13-49 RT: 0.22-0.88 AV: 37 NL: 3.84
T: ITMS + p MALDI Full ms2 384.00@cid14.00 [105.00-420.00]
Tebipenem MSMS
10 µM-1 mg/ml matrix
298.08
[M-86+H]+
Relative Abundance
m/z
384.08
356.08
340.08
312.17
280.08
254.08
366.17
270.08
322.08
295.08
173.08
141.08
151.92
121.17
177.25
196.00
213.17
220.25
238.25
251.17
266.92
308.92
338.17
353.00
369.75
401.42

Figure S2e

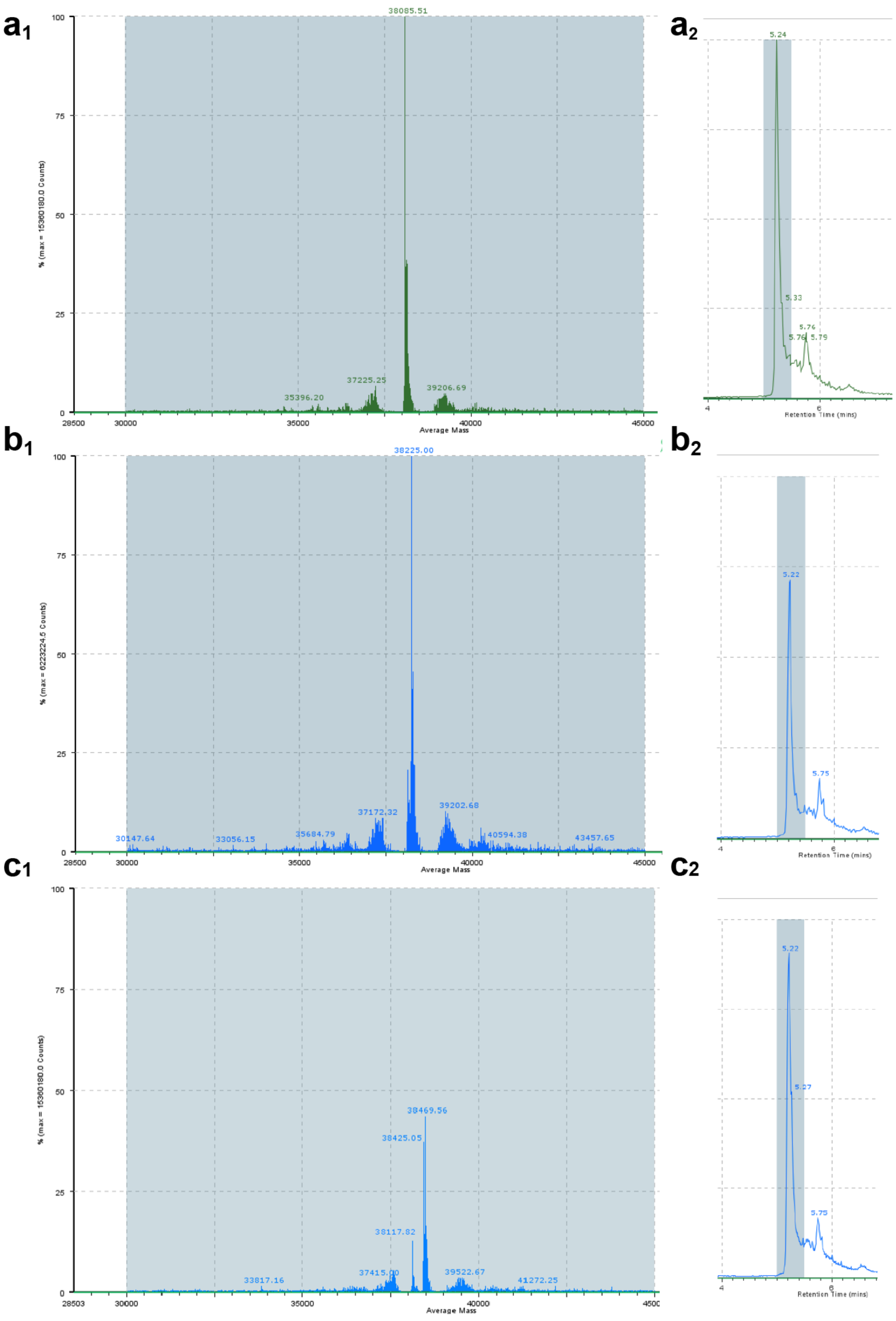

Figure S3 ($a_{1\text{-}2}$, $b_{1\text{-}2}$,$c_{1\text{-}2}$)

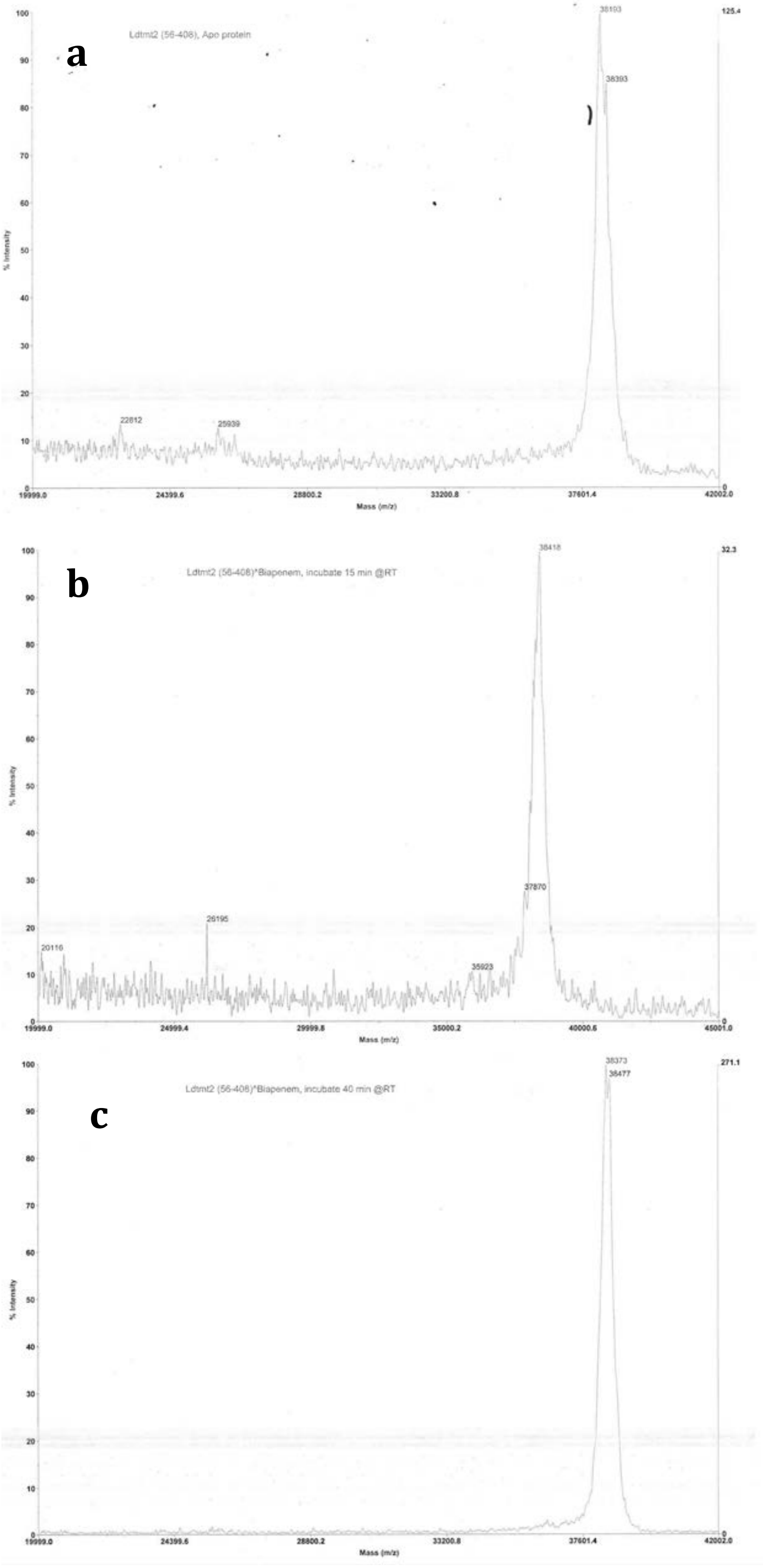
a
Ldtmt2 (56-408), Apo protein
38193
38393
125.4
22812
25939
% Intensity
Mass (m/z)
19999.0
24399.6
28800.2
33200.8
37601.4
42002.0
b
Ldtmt2 (56-408)*Biapenem, incubate 15 min @RT
38418
32.3
37870
26195
20116
35923
% Intensity
Mass (m/z)
19999.0
24999.4
29999.8
35000.2
40000.6
45001.0
c
Ldtmt2 (56-408)*Biapenem, incubate 40 min @RT
38373
38477
271.1
% Intensity
Mass (m/z)
19999.0
24399.6
28800.2
33200.8
37601.4
42002.0

. **Figure S4**

d

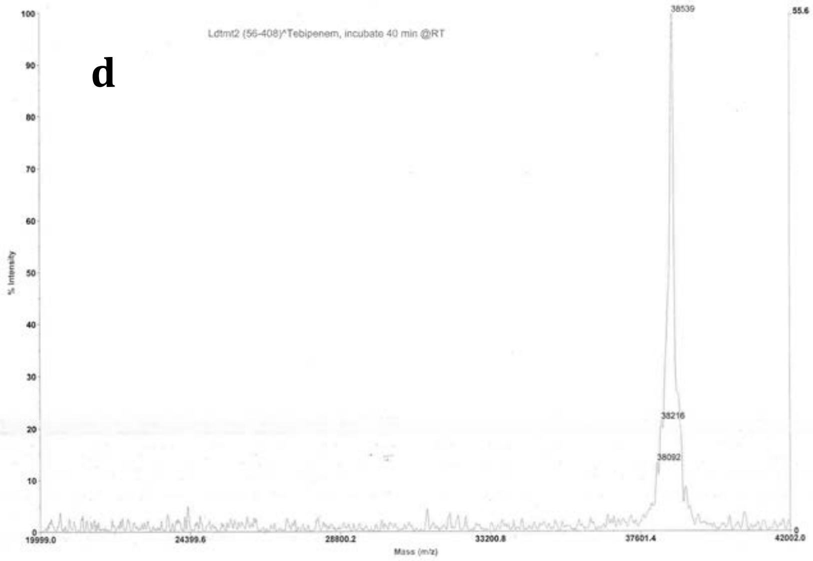

e

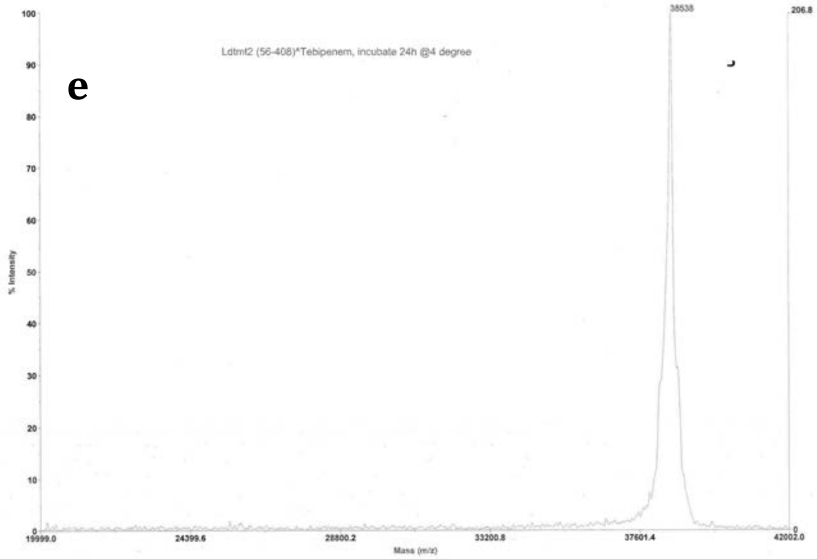

Figure S4 (cont.)
